# “Attacking” the Gut–Brain Axis with Psychobiotics: An Umbrella Review of Depressive and Anxiety Symptoms

**DOI:** 10.3390/ph19010156

**Published:** 2026-01-15

**Authors:** Alberto Souza Sá Filho, Tatiane Bastos Souza, José Luís Rodrigues Martins, Gunnar P. H. Dietz, Katia Flávia Fernandes, Stone de Sá, Pedro Augusto Inacio, Iransé Oliveira-Silva, Gustavo Pedrino, Vicente Aprigliano, Gaspar R. Chiappa, James Oluwagbamigbe Fajemiroye

**Affiliations:** 1 Graduate Program in Pharmaceutical Sciences, Evangelical University of Goiás, Anápolis 75080-000, Brazil; taatianebastos@gmail.com (T.B.S.); jose.martins@docente.unievangelica.edu.br (J.L.R.M.); katiaffernandes@ufg.br (K.F.F.); stone.sa@docente.unievangelica.edu.br (S.d.S.); jamesfajemiroye@ufg.br (J.O.F.); 2 Graduate Program in Human Movement and Rehabilitation, Evangelical University of Goiás, Anápolis 75080-000, Brazil; pedroqinacio@gmail.com (P.A.I.); iranse.silva@unievangelica.edu.br (I.O.-S.); gaspar.chiappa@gmail.com (G.R.C.); 3 Georg-August-Universität Göttingen Medical School, D-37075 Göttingen, Germany; Gunnar.Dietz@medizin.uni-goettingen.de; 4 Institute of Biological Sciences, Federal University of Goiás, Goiás 74605-010, Brazil; pedrino@ufg.br; 5 Escuela de Ingeniería de Construcción y Transporte, Pontificia Universidad Católica de Valparaíso, Avda Brasil 2147, Valparaíso 2362804, Chile; 6 Faculty of Health Sciences, Universidad Autónoma de Chile, Providencia 7500912, Chile

**Keywords:** probiotics, prebiotics, depressive disorder, anxiety disorders, gut–brain axis

## Abstract

**Background/Objectives**: This umbrella review critically evaluates the available evidence on psychobiotics for depressive and anxiety symptoms, emphasizing methodological quality, consistency of findings, and persistent gaps in the literature. **Methods**: A comprehensive search was conducted across PubMed/MEDLINE, Scopus, Web of Science, SciELO, Cochrane, and EBSCO (May–June 2025) to identify systematic reviews with meta-analyses of randomized controlled trials examining probiotic, prebiotic, and synbiotic interventions in adults with depressive and/or anxiety symptoms or diagnoses. Two reviewers independently screened studies, extracted data, and evaluated methodological quality using AMSTAR-2. Additional bibliometric, conceptual, and psychometric features were mapped, including geographical origin, publication timeline, scale distribution, and citation-based connectivity. **Results**: Thirty systematic reviews and meta-analyses were included. Methodological quality was predominantly moderate, low, or critically low in 76.6% of reviews. Probiotic interventions demonstrated consistent benefits for MDD (SMD = −0.50 [95% CI: −0.58 to −0.42], *p* = 0.0001). However, findings for anxiety were markedly inconsistent, despite the modest improvements in specific subgroups (SMD = −0.19 [95% CI: −0.28 to −0.10]; *p* < 0.01). Prebiotics for MDD interventions showed limited positive results (SMD = −0.25 [95% CI: −0.47 to −0.03]; *p* = 0.03). For anxiety, the effects are inconclusive (SMD = −0.07 [95% CI: −0.30 to 0.10]; *p* = 0.18). Evidence for synbiotics was scarce. Citation-mapping revealed a fragmented and unevenly connected evidence base. **Conclusions**: The current evidence suggests that probiotics may confer beneficial effects on depressive and anxiety symptoms; however, the same cannot be said for prebiotics and synbiotics. Evidence for the efficacy of prebiotics and synbiotics to treat depression and anxiety is still insufficient or heterogeneous. Registration: CRD420251164884.

## 1. Introduction

Over the past two decades, the growing interest in the relationship between gut microbiota and mental health has driven research into psychobiotic-based interventions as adjunct therapeutic strategies for disorders such as depression and anxiety [1,2,3,4,5,6]. Defined by Dinan et al. [7] as “live organisms which, when ingested in adequate amounts, confer health benefits in patients with psychiatric disorders,” this perspective has emerged as a promising alternative or complement to conventional pharmacotherapy. Unlike traditional antidepressants, psychobiotics act not only on central neurotransmitter systems but also modulate peripheral pathways, including inflammation, oxidative stress, and intestinal barrier integrity [5,8,9,10,11,12]. Psychobiotics include probiotics (beneficial microbes), prebiotics (fibers that feed them), and synbiotics (their combined, mutually supportive pair) [13].

Additionally, their ability to influence the hypothalamic–pituitary–adrenal (HPA) axis and to promote resilience to stress-related disorders has been demonstrated in both preclinical and clinical contexts [14,15,16]. Moreover, their favorable safety profile and potential synergistic effects with antidepressants suggest that psychobiotics may be particularly beneficial in cases of treatment-resistant depression or among patients with low tolerance to pharmacological agents [17]. The integration of psychobiotics into conventional care models represents a step forward toward more personalized, gut-targeted approaches to mental health. For instance, the study conducted by Akkasheh et al. [1] reported significant improvements in depression scores (Beck Depression Inventory-BDI), insulin levels, HOMA-IR, and C-reactive protein (hs-CRP) following eight weeks of supplementation with *Lactobacillus* and *Bifidobacterium* strains.

In response to this growing movement, the scientific literature has been marked by a substantial increase in the number of systematic reviews and meta-analyses over the years [5,8,9,11,16,17,18,19,20,21,22,23,24,25,26,27,28,29,30,31,32,33,34,35,36,37,38,39,40,41,42,43,44,45,46,47,48,49,50,51,52,53,54]. Many of these focus specifically on clinically diagnosed populations [28,30,31,32,35,38,42,43,46], while others include non-clinical or subclinical samples [9,18,19,29,30,36,37,41,55]. Although this expanding body of publications reflects the recognition of the therapeutic potential of psychobiotics, i.e., the current state of the art, it also introduces considerable complexity. Reviews often diverge regarding the efficacy of interventions, the methodological rigor of the included trials [23], the selection of evaluated outcomes (clinical scales [47], self-reported symptoms, and biomarkers [43]), and the characterization of the strains employed [5,22,29,43]. Furthermore, key elements such as intervention duration, formulation type [9,46,47,52], and even stratification by biological sex or age have been inconsistently addressed or overlooked in most available reviews. This heterogeneity hampers the generalizability of findings and limits the clinical applicability of the existing evidence.

Thus, despite the multiplicity of published reviews, there remains a lack of integrative and comparative analyses across studies. Few approaches have critically systematized the findings [21,23], identified contradictions, or, most importantly, exposed the gaps that persist in the literature. Consequently, the field remains open to a study that not only consolidates the current state of the art but also rigorously and critically evaluates the methodological quality and overall landscape of existing reviews. Therefore, the present study aims to systematically compile, analyze, and compare the main systematic reviews with meta-analyses on the use of psychobiotics in the treatment of depressive and anxiety disorders or symptom levels, emphasizing the chronological evolution of the evidence, major methodological inconsistencies, and, above all, the gaps that must still be addressed by future clinical trials and more robust reviews.

## 2. Materials and Methods

### 2.1. Experimental Approach and Protocol Register

The systematic search for systematic reviews, as well as the construction of the present review, was partially conducted in accordance with the Preferred Reporting Items for Systematic Reviews and Meta-Analyses (PRISMA) guidelines [56] and fully assessed for methodological quality using the AMSTAR 2 tool (a measurement tool to assess systematic reviews). This study was registered in the PROSPERO database under the number CRD420251164884.

### 2.2. Study Design

Only systematic reviews with meta-analyses of randomized controlled trials (RCTs) were eligible for inclusion. To be included, the trials had to investigate the effects of psychobiotic interventions, such as probiotics, prebiotics, or synbiotics, administered with the aim of improving symptoms of depression and/or anxiety and/or stress. Secondarily, changes in biological markers, such as neuroinflammatory indicators, were also examined. Only studies conducted in humans and published in English were considered.

The following studies were excluded: (1) observational studies, standalone systematic reviews, case reports, and preclinical studies involving animals or in vitro models; (2) studies assessing gut microbiota modulation without considering depression or anxiety symptoms as primary or secondary outcomes; (3) studies using antibiotics or fecal microbiota transplantation as the main intervention; (4) studies including samples with other psychiatric disorders, such as schizophrenia, psychosis, attention-deficit/hyperactivity disorder, or post-traumatic stress disorder, as the primary focus of investigation; and (5) studies focusing on special conditions, such as pregnancy or the postpartum period, were also excluded.

### 2.3. Participants

Systematic reviews with meta-analyses that investigated individuals diagnosed with depressive and/or anxiety disorders were included, based on standardized diagnostic criteria, such as those outlined in the *Diagnostic and Statistical Manual of Mental Disorders* or the International Classification of Diseases. However, meta-analyses that included mixed populations, provided they also enrolled participants diagnosed with depression and/or anxiety, were retained but classified as secondary evidence and did not impact the primary analysis. This category included studies involving healthy individuals, students, or clinical populations with systemic or mental comorbidities not specifically related to mood or anxiety disorders or clinically affected by depression and anxiety. Additionally, only studies involving adults were considered. No restrictions were applied regarding sex, ethnicity, or disease duration.

### 2.4. Study Selection

Between May and June 2025, two reviewers (JOF and ASF) independently conducted a comprehensive literature search using the PubMed/MEDLINE, Web of Science, Scopus, Scielo, EBSCO, and Cochrane Database electronic databases. The search was restricted to studies involving human subjects and published in English up to September 2025. The reference lists of relevant studies (other sources) were also manually examined to identify additional eligible articles. The complete search strategy combined terms using Boolean operators across the different databases, as presented in Table S1 (Supplementary Materials). Additionally, the file related to the extraction of all databases was deposited in The Open Science Framework—OSF (https://osf.io/fq5c9/overview; access on 7 January 2026).

In general, we combined the following keywords, which varied in their inclusion format depending on the database: (psychobiotics OR probiotics OR prebiotics OR synbiotics) AND (depressive OR depressive disorder OR anxiety) AND meta-analysis.

### 2.5. Primary and Secondary Specific Outcomes

The primary outcomes of this review included changes in symptoms of depression and/or anxiety, assessed through validated psychometric instruments. All scales available in the different meta-analyses were included, such as, the Beck Depression Inventory (BDI), the Depression, Anxiety and Stress Scales (DASS), the Hospital Anxiety and Depression Scale (HADS), the Montgomery–Åsberg Depression Rating Scale (MADRS), the Hamilton Depression Rating Scale (HAM-D), the Patient Health Questionnaire-9 (PHQ-9), the Beck Anxiety Inventory (BAI), the State–Trait Anxiety Inventory (STAI), the Perceived Stress Scale (PSS), the Hamilton Anxiety Rating Scale (HAM-A), and the Generalized Anxiety Disorder-7 (GAD-7). Changes in these scores from baseline to post-intervention were analyzed as indicators of improvement in mood and anxiety symptoms.

As secondary outcomes, biological markers and physiological mechanisms related to gut–brain axis modulation were considered, aiming to explore the biological basis of psychobiotic effects, although they were not included in the statistical analysis. These markers included the following: (a) biomarkers related to neurotransmitters, such as serum or plasma levels of serotonin, dopamine, gamma-aminobutyric acid (GABA), and noradrenaline, as well as tryptophan concentration and the kynurenine/tryptophan ratio; (b) metabolites produced by the gut microbiota, particularly short-chain fatty acids (SCFAs), including butyrate, propionate, and acetate; (c) inflammatory and immunological markers, such as interleukin-6 (IL-6), tumor necrosis factor alpha (TNF-α), C-reactive protein (CRP), and interleukin-10 (IL-10); (d) endocrine responses to stress, especially cortisol levels (in saliva, serum, or urine) and measures of hypothalamic–pituitary–adrenal (HPA) axis regulation; and, when available, data on gut microbiota composition.

### 2.6. Extraction of Bibliometric, Conceptual, and Psychometric Characteristics

In addition to the traditional methodological variables, an expanded set of bibliometric and psychometric characteristics was systematically extracted to map temporal, geographical, and conceptual patterns across the included studies.

Information regarding the geographical origin of each study (country and continent of conduct) was recorded to identify the spatial distribution of scientific production and potential regional asymmetries. Subsequently, all studies were organized according to their chronological order of publication, from which a timeline was constructed to illustrate the historical evolution of the evidence and periods of increased or reduced research activity. To characterize the conceptual structure of the literature, a citation-based connectivity map was generated, capturing the structural relationships among the included studies, revealing variations in citation density and the strength of inter-study links. This mapping approach elucidates how the evidence base is organized, identifying central nodes (hubs) and more peripheral contributions to the progression of research on psychobiotics.

Additionally, standardized extraction of the psychometric scales used in each study was performed, covering instruments for the assessment of depression, anxiety, stress, quality of life, clinical symptoms, and other psychological domains. All scales were categorized according to their purpose, psychometric properties, and frequency of use, enabling comparative synthesis across instruments and identification of methodological convergences among the included studies.

### 2.7. Risk-of-Bias Assessments

The methodological quality of the included systematic reviews was assessed using the AMSTAR 2 instrument. Two assessors independently carried out this process (JOF and ASF). AMSTAR 2 comprises 16 items addressing key methodological aspects such as the presence of a pre-registered protocol, adequacy of inclusion criteria, search strategy, assessment of risk of bias in primary studies, and appropriateness of statistical analyses. This tool does not generate a final numerical score but rather classifies the overall confidence in the review’s findings as high, moderate, low, or critically low, allowing for a more qualitative interpretation of the methodological robustness of each evaluated study [57].

### 2.8. Inter-Rater Agreement

The screening of titles, abstracts, and full texts was independently performed by two reviewers, strictly following the predefined inclusion and exclusion criteria. To assess the reliability of the selection process, Cohen’s Kappa coefficient was calculated, which measures the degree of agreement beyond that expected by chance. The coefficient was obtained from the proportion of observed agreement (P_o_) and the proportion of expected agreement by chance (P_e_) according to the following formula:

$$\kappa \;=\;\frac{Po-Pe}{1-Pe}$$

This procedure is recommended in systematic reviews and meta-analyses, as it ensures greater transparency and reproducibility in study selection while minimizing potential biases derived from subjective judgments.

### 2.9. Statistical Analysis

Statistical analyses were conducted by extracting effect sizes as SMDs accompanied by their 95% confidence intervals (95%CI). Between-study variability was quantified using the I^2^ statistic. The overall pooled effect was estimated using a random-effects model. Additionally, sensitivity analyses were carried out to assess the robustness of the findings by sequentially excluding studies judged to have a high variability. In this procedure, each meta-analytic estimate was sequentially removed, and the random-effects model was recalculated to determine whether any single study exerted disproportionate influence on the summary effect. Stability of the pooled SMD across iterations was taken as evidence that the findings were not driven by isolated data points or outlier effect sizes.

To assess the robustness of the pooled SMD, a leave-one-out sensitivity analysis was performed, in which the random-effects model was recalculated sequentially after removing each meta-analysis individually.

Cochran’s Q test was used to assess the presence of heterogeneity, while the magnitude of inconsistency was quantified using the I^2^ statistic, interpreted according to established thresholds (25% low, 50% moderate, and 75% high heterogeneity). In addition, the between-study variance (τ^2^) was estimated using the DerSimonian–Laird method to account for the dispersion of true effect sizes across studies. Because substantial heterogeneity was expected due to differences in population characteristics, psychometric scales, intervention duration, probiotic strains, and study design, all pooled estimates were computed using random-effects models.

## 3. Results

### 3.1. Search Results and Inter-Rater Agreement

**Figure 1:**
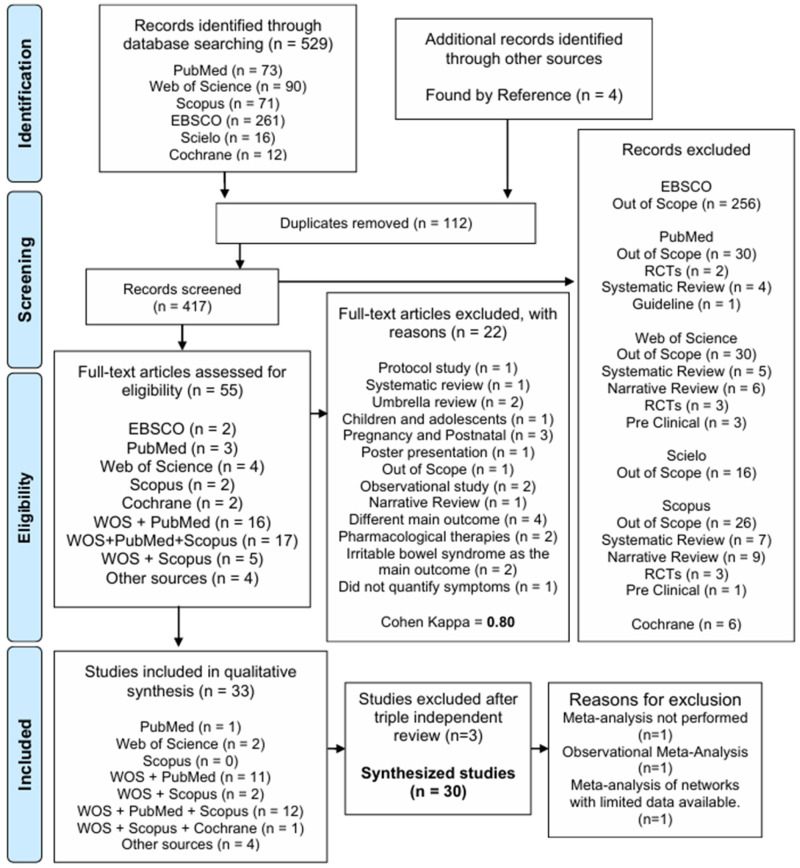
Evidence inclusion and exclusion flow.

The systematic search initially identified 529 articles related to the topic. After removing duplicates and reading by title and abstract, the screening observed 55 systematic reviews with meta-analyses across the selected databases and additional sources. Of these, 22 were excluded for not meeting the predefined inclusion criteria (e.g., scope outside the research question or incompatible population), leaving 33 articles for full-text assessment. In the end, only 30 systematic reviews with meta-analyses remained. A list of the included articles was presented separately, with the respective databases from which they were extracted in Table S2 (Supplementary Materials). Figure 1 details the inclusion and exclusion process for the selected articles. The Supplementary Materials (Table S3) detail the reasons for exclusion.

During the screening process, a high level of consistency was observed between the two independent reviewers. Cohen’s Kappa coefficient was 0.80, a value corresponding to substantial to almost perfect agreement according to the criteria of Landis and Koch. This result confirms the robustness of the selection process and minimizes the risk of inappropriate inclusion or exclusion of studies in the present meta-analysis.

The included meta-analyses showed considerable methodological heterogeneity, both regarding search strategies and eligibility criteria, population, and strains used in the intervention, as well as in relation to the outcomes analyzed. The number of primary studies included within each meta-analysis ranged from 5 to 62, with pooled sample sizes varying from 365 to 5245 participants. Most of the reviews were published after 2020, in journals indexed primarily in the PubMed and Web of Science databases (22 meta-analyses; 66.6%), simultaneously, and the investigated populations were characterized by a diagnosis of mood and/or anxiety disorder, or healthy participants. However, a few studies added analyses for patients with other diagnoses. In our final analysis, we discarded the extraction of groups with varied diagnoses, focusing primarily on aspects of depression and anxiety.

### 3.2. Bibliometric Analysis and Characteristics of the Studies

North America accounted for three meta-analyses (9.1%), with studies from the United States [31,32] and Canada [39].

South America contributed one meta-analysis (3.0%), represented by Brazil [42], whereas in Europe contributed seven meta-analyses (21.2%), which were conducted in France [16], the United Kingdom [34,40,41,48], Germany [43], and Poland [35].

**Figure 2:**
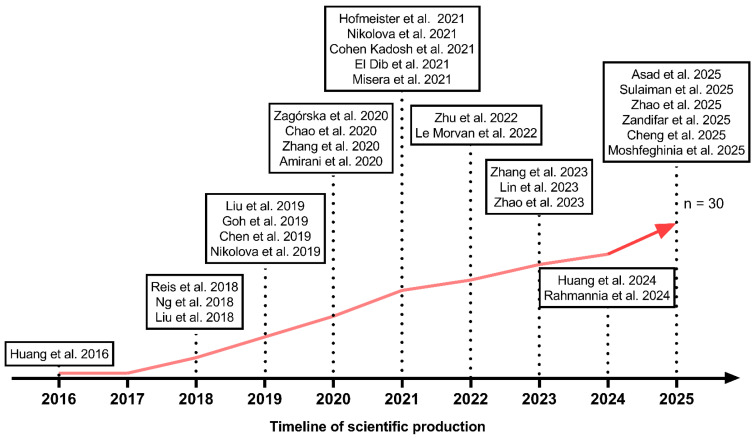
Timeline of scientific production of meta-analyses. This figure summarizes the chronological progression of scientific publications included in the review from 2016 to 2025. The timeline highlights the following studies: Huang et al. [28]; Reis et al. [31]; Ng et al. [17]; Liu et al. [30]; Liu et al. [32]; Goh et al. [33]; Chen et al. [58]; Nikolova et al. [34]; Zagórska et al. [35]; Chao et al. [36]; Zhang et al. [37]; Amirani et al. [38]; Hofmeister et al. [39]; Nikolova et al. [40]; Cohen Kadosh et al. [41]; El Dib et al. [42]; Misera et al. [43]; Zhu et al. [54]; Le Morvan et al. [16]; Zhang et al. [27]; Lin et al. [44]; Zhao et al. [45]; Huang et al. [46]; Rahmannia et al. [47]; Asad et al. [48]; Sulaiman et al. [49]; Zhao et al. [50]; Zandifar et al. [51]; Cheng et al. [52]; Moshfeghinia et al. [53].

**Figure 3:**
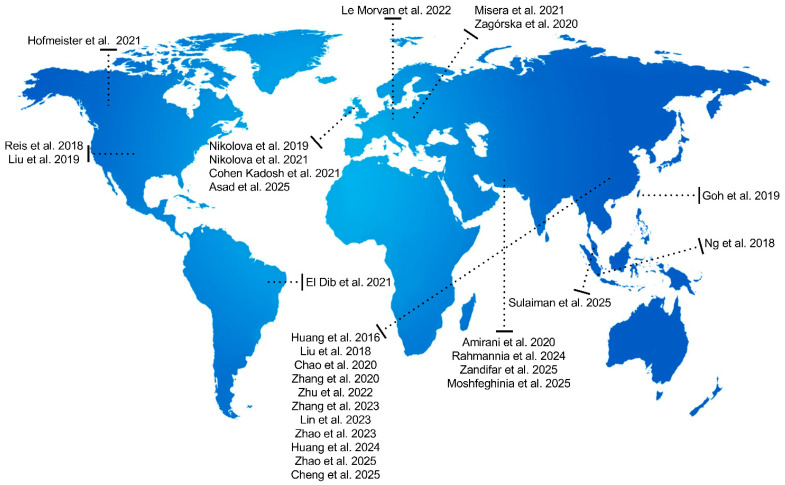
Global distribution of the studies included in the review. This figure illustrates the geographical distribution of the 30 studies included in the review, mapped according to the countries where the research was conducted. The studies span multiple regions, demonstrating the global interest in the topic. Publications are distributed across North America (Reis et al. [31]; Liu et al. [32]; Hofmeister et al. [39]), Europe (Nikolova et al. [34]; Nikolova et al. [40]; Chao et al. [36]; Zagórska et al. [35]; Misera et al. [43]; Cohen Kadosh et al. [41]; Le Morvan et al. [16]; Asad et al. [48]), South America (El Dib et al. [42]), Asia (Huang et al. [28]; Liu et al. [30]; Zhang et al. [37]; Zhu et al. [54]; Zhang et al. [27]; Lin et al. [44]; Zhao et al. [45]; Huang et al. [46]; Zhao et al. [50]; Cheng et al. [52]), Southeast Asia (Goh et al. [33]; Ng et al. [17]), and the Middle East (Amirani et al. [38]; Rahmannia et al. [47]; Zandifar et al. [51]; Moshfeghinia et al. [53]; Sulaiman et al. [49]).

Asia showed the highest concentration of research, with 20 meta-analyses (60.6%), including China [27,28,30,36,37,44,46,50,52,54,58], Singapore [33], Malaysia [17], and Korea [29]. Finally, the Middle East contributed six meta-analyses (18.2%), from Iran [38,47,51,53,59] and Saudi Arabia [49]. Figure 2 presents the timeline of scientific production. Figure 3 presents the global distribution of the studies included in the review.

This map illustrates the geographical distribution of all clinical trials included in the systematic review. The studies were conducted across North America, South America, Europe, Asia, Africa, and Oceania, highlighting the multinational and heterogeneous nature of the available evidence.

**Figure 4:**
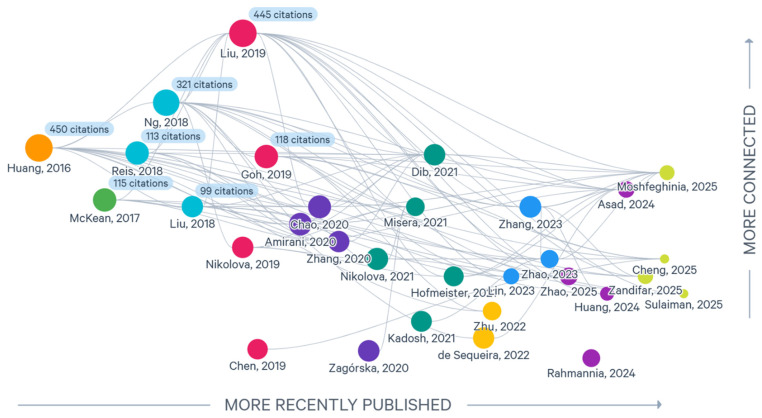
Citation-based connectivity map of the included studies. This figure depicts the citation network among the 30 included studies, illustrating how research groups have built upon one another over time. Each node represents an individual study, positioned along a horizontal axis indicating recency of publication (more recent to the right) and a vertical axis indicating degree of connectedness, measured by incoming and outgoing citation links (more connected toward the top). Huang et al. [28]; McKean et al. [29]; Reis et al. [31]; Ng et al. [17]; Liu et al. [30]; Liu et al. [32]; Goh et al. [33]; Nikolova et al. [34]; Zagórska et al. [35]; Chao et al. [36]; Zhang et al. [37]; Amirani et al. [38]; Hofmeister et al. [39]; Nikolova et al. [40]; Cohen Kadosh et al. [41]; El Dib et al. [42]; Misera et al. [43]; Zhu et al. [54]; Le Morvan et al. [16]; Zhang et al. [27]; Lin et al. [44]; Zhao et al. [45]; Huang et al. [46]; Rahmannia et al. [47]; Asad et al. [48]; Sulaiman et al. [49]; Zhao et al. [50]; Zandifar et al. [51]; Cheng et al. [52]; Moshfeghinia et al. [53]; Chen et al. [58].

Figure 4 presents the citation-based connectivity map of the meta-analyses included in this review. The visualization illustrates the extent to which individual papers share similar reference lists, which reflect patterns of methodological overlap rather than scientific influence in a causal sense. Accordingly, high citation density in this context indicates that two papers draw on a comparable pool of primary studies, often because they employed similar search strategies or targeted similar publication periods.

From this perspective, the high connectivity observed for papers such as Liu et al. [32] and Huang et al. [28] should not be over-interpreted as evidence of conceptual influence or foundational status. Instead, their large number of shared references likely reflects the fact that these reviews included broad search windows and extensive primary-study lists, increasing the probability of overlap with other meta-analyses. So, high connectivity among more recent papers may indicate redundancy; when a new meta-analysis cites nearly the same set of primary studies already synthesized in earlier work, this suggests that its analytical contribution may be limited.

In contrast, more peripheral papers (e.g., Zhang et al. [27], Zhao et al. [50], and Cheng et al. [52]) that display low inter-study connectivity may be incorporating newer datasets, diverging populations, or more selective inclusion criteria—features that can enhance their added value despite having fewer shared citations.

Thus, our citation-based connectivity map provides a structural overview of how similarly (or differently) the included meta-analyses assembled their evidence bases. This distinction is important to avoid conflating methodological overlap with scientific impact.

The map reveals a clear hierarchical structure: a small number of highly cited, conceptually influential studies anchor the network, intermediate studies provide bridging links, and emerging publications expand the field in more specialized directions.

**Figure 5:**
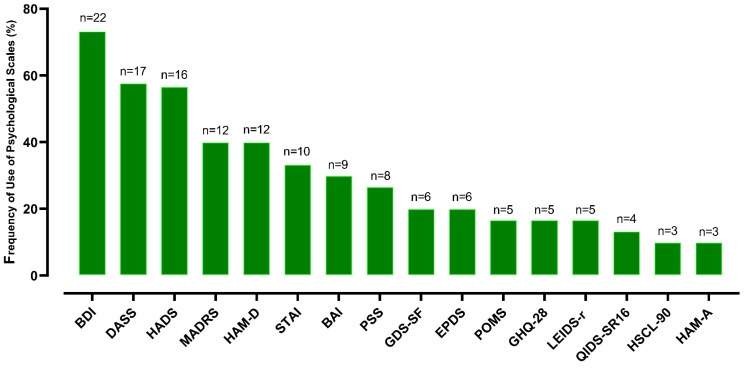
Distribution of psychological assessment instruments used across the included studies. **Abbreviations:** BDI = Beck Depression Inventory; DASS = Depression, Anxiety and Stress Scales; HADS = Hospital Anxiety and Depression Scale; MADRS = Montgomery–Åsberg Depression Rating Scale; HAM-D = Hamilton Depression Rating Scale; STAI = State–Trait Anxiety Inventory; BAI = Beck Anxiety Inventory; PSS = Perceived Stress Scale; GDS-SF = Geriatric Depression Scale—Short Form; EPDS = Edinburgh Postnatal Depression Scale; POMS = Profile of Mood States; GHQ-28 = General Health Questionnaire—28 items; LEIDS-r = Leiden Index of Depression Sensitivity—Revised; QIDS-SR16 = Quick Inventory of Depressive Symptomatology—Self-Report (16 items); HSCL-90 = Hopkins Symptom Checklist—90 items; and HAM-A = Hamilton Anxiety Rating Scale.

The included reviews employed a wide range of psychometric instruments, reflecting the conceptual and methodological heterogeneity of the field. The scales identified encompassed assessments of depressive symptoms, anxiety, perceived stress, mood, general psychological distress, and well-being. A predominance of traditional measures of depression and emotional disorders was observed, alongside instruments tailored to specific clinical populations or sensitive periods, such as pregnancy and the postpartum phase. This diversity highlights both the complexity of the constructs under investigation and the lack of standardization across studies, which challenges direct comparisons and underscores the need for analytical approaches that account for conceptual and sensitivity differences among instruments (Figure 5).

Finally, according to the initial extraction plan, we present the number of participants, age and population status, intervention (mono-strain or multi-strain), and duration, as well as the outcomes reported. General characteristics of the 33 eligible meta-analyses are summarized in Table 1.

Probiotics accounted for the largest proportion, with 30 studies (100%) examining their effects on psychological outcomes [16,17,27,28,29,30,31,32,34,35,36,37,38,39,40,41,42,43,44,45,46,47,48,49,50,51,52,53,54]. Prebiotics represented seven studies (23.3%), reflecting a smaller body of evidence focusing on isolated modulation of fermentable fibers [27,32,39,41,45,51,53]. Synbiotics were evaluated in four studies (13.3%), indicating more limited interventions [27,39,45,51]. Although several studies appeared in more than one category due to overlapping formulations or multifactorial designs, these proportions provide a clear depiction of the relative emphasis placed on each intervention type within the current evidence base.

### 3.3. Summary and Guidance on the Results

#### 3.3.1. General Information and Problems Encountered

To minimize analytical heterogeneity and avoid distorting the behavior of the pooled estimates, the results were synthesized separately for depression scores and anxiety scores. This decision was based on the markedly larger number of studies evaluating the effects of probiotics on depressive symptoms compared to those assessing anxiety, which could have disproportionately influenced the overall summary effect if analyzed jointly. In addition, corrections were applied to the directionality of the reported outcomes. Specifically, several meta-analyses presented positive SMDs to indicate improvements associated with probiotic supplementation. Because the present synthesis required all outcomes to be plotted on a common axis for comparability, these SMDs were systematically reoriented to negative values. This restandardization ensured conceptual consistency, whereby negative values uniformly reflected symptom improvement and prevented misleading interpretations that could arise from mixing opposing effect directions within the same analytical framework.

When individual studies reported mean differences rather than SMD, the values were systematically converted to SMD to ensure comparability across meta-analyses and maintain scale-independent interpretation. All synthesis procedures were conducted using random-effects models to account for substantial between-study heterogeneity.

We elected to exclude the meta-analysis by McKean et al. [29] from the primary synthesis due to its limited relevance and the predominance of healthy participants in its pooled sample. Additionally, part of the evidence incorporated in that meta-analysis originated from a study involving stressed individuals, which was subsequently retracted and removed from the scientific record.

A specific methodological inconsistency was identified in one of the included meta-analyses, Nikolova et al. [34]. In their review, the authors report having included three clinical trials. However, one of these trials, Kazemi et al. [60], published in *Clinical Nutrition*, appears with effect size values that are statistically implausible. Specifically, Nikolova et al. [34] present an estimated effect of SMD = 1.995 (95% CI: 1.437 to 2.552) for this single study. As a consequence, the pooled effect of probiotic supplementation was deemed ineffective in their meta-analysis (SMD = 0.826, 95% CI: −0.527 to 2.178; *p* = 0.231; I^2^ = 94.7%), driven almost entirely by this anomalous estimate.

In contrast, multiple other meta-analyses evaluating the same body of evidence report substantially different, and more coherent, effect size estimates for Kazemi et al. [60]. For example, Zagórska et al. [35] report an individual effect size of SMD = −0.70 (95% CI: −1.17 to −0.23) for this study, which is opposite in direction and markedly smaller in magnitude. Similar estimates are found in several more recent meta-analyses, further reinforcing the likelihood of a data extraction or coding error in Nikolova et al. [34].

Therefore, it was necessary to first correct the directionality of the effects (i.e., negative values indicating improvement in depressive symptoms) and subsequently re-estimate the effect size to ensure consistency with the broader literature. This adjustment was essential to avoid propagation of erroneous data and to maintain the methodological rigor of the present synthesis.

Finally, it is important to note that, despite the inclusion of 30 meta-analyses, in some cases, certain studies were not incorporated into the final synthesis because the interventions were not stratified (Zandifar et al. [51]). As a result, evidence from probiotics, prebiotics, and synbiotics was pooled within the same analysis without performing subgroup assessments.

#### 3.3.2. Synthesis Strategy and Selection of Effect Estimates

The synthesis of results among the included meta-analyses required careful consideration of how SMDs were selected and extracted, given the substantial heterogeneity in study populations, symptom severity, and outcome assessment instruments. When available, subgroup-specific SMDs were preferentially extracted for analyses targeting depressive or anxiety symptoms, particularly when meta-analyses reported stratified estimates based on clinically diagnosed populations, symptom severity, or specific psychometric instruments. This approach aimed to enhance clinical interpretability and reduce dilution of effects that could arise from pooling heterogeneous populations, such as healthy individuals and participants without clinically relevant symptoms.

In contrast, global SMDs were retained when subgroup stratification was either not clearly defined, not methodologically justified, or when the study population was already homogeneous with respect to depressive or anxiety disorders. In several cases, global estimates were also preferred when further stratification would have substantially reduced the number of contributing studies, thereby compromising statistical stability and interpretability. For meta-analyses reporting multiple subgroup estimates based on different psychometric scales, the SMD derived from the most prevalent instrument was selected to ensure consistency and comparability among analyses.

This decision-making process was applied systematically and transparently among all included meta-analyses, with detailed justifications provided in Supplementary Table S5.

#### 3.3.3. Qualitative Summary of the Results

Among the 30 included meta-analytic estimates, 22 studies using probiotics for intervention purposes demonstrated the most consistent evidence of benefit for depressive and anxiety symptoms. For depressive outcomes, 92.0% of the probiotic meta-analytic estimates reported statistically significant reductions in symptom severity, although the effects appear somewhat inconsistent when analyzing other scales, such as MADRS. Positive effects were observed in the majority of studies [16,17,27,31,32,33,35,36,39,40,42,43,44,45,46,48,49,50,51,52,53,54].

Conversely, two meta-analytic studies (8.0%) produced non-significant effects, including the studies by Liu et al. [30] and Reis et al. [31], as well as two smaller analyses in which confidence intervals crossed the null. Importantly, no probiotic meta-analysis showed a significant effect favoring the control group, reinforcing the directional consistency of findings. The overall pooled effect reflects a moderate and clinically meaningful reduction in symptoms.

For anxiety outcomes, 58.3% of the probiotic estimates showed significant improvements, driven by studies such as Moshfeghinia et al. [53], Zandifar et al. [51], Asad et al. [48], Zhao et al. [45], El Dib et al. [42], Zhang et al. [37], and Liu et al. [32]. Non-significant findings were reported by Zagórska et al. [35], Cohen Kadosh et al. [41], Le Morvan et al. [16], Chao et al. [36], and Reis et al. [31], although none demonstrated detrimental effects. The overall pooled effect reflects a small-to-moderate anxiolytic benefit.

In contrast, evidence for prebiotics was less consistent. Among the four meta-analytic estimates for depressive symptoms, two (50%), specifically Moshfeghinia et al. [53] and Hofmeister et al. [39], identified statistically significant improvements, while Zhang et al. [27] and Liu et al. [32] reported non-significant effects. Still, the pooled effect suggests a small but statistically reliable protective effect. Logically, this information should be interpreted with caution.

For prebiotics and anxiety, neither included study (Zhao et al. [45] and Liu et al. [32]) demonstrated significant improvements, yielding a non-significant pooled estimate. No study favored the control group, but the consistency of null results suggests insufficient evidence for anxiolytic efficacy of isolated prebiotic supplementation.

Finally, a total of four meta-analyses evaluated the effects of synbiotic interventions on depressive/anxiety symptoms. Among these, only one study assessed the effects of synbiotics on depression, and one evaluated their effects on anxiety, reporting a favorable effect. The other studies did not provide estimable or clinically interpretable effects for synbiotics, thereby limiting quantitative synthesis.

The study by Hofmeister et al. [39] reported an estimated effect exclusively in healthy populations, without including clinical samples; consequently, its findings were not suitable for integration into the main analysis. Similarly, Zhang et al. [27] evaluated only a single primary study and did not report an estimated effect size for synbiotics. Furthermore, the included study investigated a combined intervention consisting of multi-strain probiotics plus fluoxetine, preventing the isolation of synbiotic-specific effects.

Considering the individual outcomes analyzed, we cannot estimate the true effect of synbiotics on symptoms of depression (Moshfeghinia et al. [53]: SMD= −1.25 [95%CI −1.91 to −0.58]; *p* < 0.001) and anxiety (Zhao et al. [45]: −0.71 [95%CI −1.04 to −0.38]; *p* < 0.01).

#### 3.3.4. Effects of Probiotic Use on Symptoms of Depression

A total of 22 meta-analytic estimates reporting depressive symptom outcomes were synthesized, following a structured subgroup strategy restricted to patients with MDD or clinical conditions in which depressive symptoms were a primary or secondary diagnostic component. This approach ensured that the pooled effect specifically reflected populations in whom depressive symptomatology was clinically relevant, rather than aggregated data from heterogeneous or asymptomatic samples.

The overall pooled effect demonstrated a moderate and statistically significant reduction in depressive symptoms in favor of probiotics (SMD = −0.50; 95% CI = −0.58 to −0.42, *p* = 0.0001). This magnitude corresponds to a clinically meaningful improvement and was consistently observed among the subgroup of individuals with clinically defined depressive symptomatology.

Inspection of individual studies revealed that several estimates fell within the “large” effect range (e.g., Moshfeghinia et al. [53], Zagórska et al. [35], and Hofmeister et al. [39]), while most studies demonstrated small-to-moderate effects. Importantly, the directionality of the effect remained uniform across all included analyses. Even studies with non-significant effects (e.g., Liu et al. [30]) contributed consistently to the overall trend to alleviate depression. Overall, the synthesized evidence demonstrates that probiotic supplementation is associated with a moderate reduction in depressive symptoms among individuals with MDD or comorbid depressive conditions.

**Figure 6:**
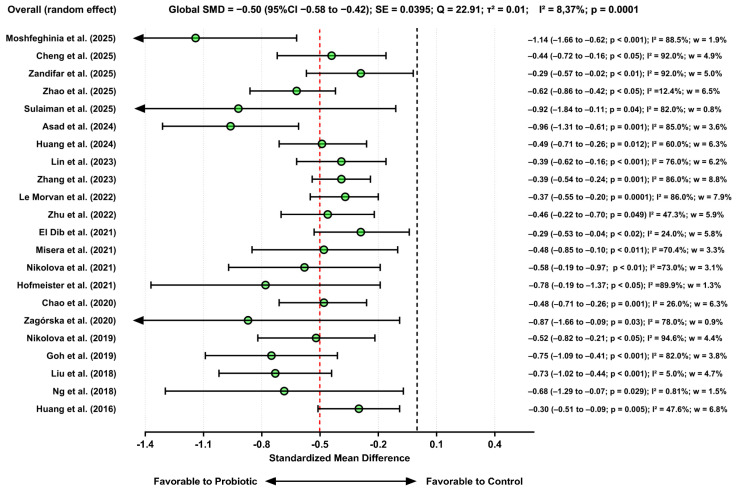
Forest plot summarizing the SMDs for the effects of probiotics on depressive symptom outcomes among 22 meta-analytic estimates. Individual study estimates are represented by circles, with horizontal lines indicating their respective confidence intervals; the size of each marker reflects the study’s relative weight in the random-effects model. Studies on probiotics and depression include: Huang et al. [28]; Ng et al. [17]; Liu et al. [32]; Goh et al. [33]; Nikolova et al. [34]; Zagórska et al. [35]; Chao et al. [36]; Hofmeister et al. [39]; Nikolova et al. [40]; El Dib et al. [42]; Misera et al. [43]; Zhu et al. [54]; Le Morvan et al. [16]; Zhang et al. [27]; Lin et al. [44]; Huang et al. [46]; Asad et al. [48]; Sulaiman et al. [49]; Zhao et al. [50]; Zandifar et al. [51]; Cheng et al. [52]; Moshfeghinia et al. [53].

The sensitivity analysis (leave-one-out) demonstrated a high degree of stability in the estimates. Excluding any individual study resulted in only marginal changes in the pooled SMD, which ranged from −0.50 to −0.44. Notably, none of the recalculated models yielded confidence intervals crossing zero, indicating that the effect remained statistically significant under all evaluated conditions. Studies with more extreme SMDs (e.g., Moshfeghinia et al. [53], SMD = −1.22) or smaller effect sizes (e.g., Liu et al. [30], SMD = −0.12) did not exert disproportionate influence on the overall result. The minimal variation observed (<0.04 points in the pooled SMD) indicates that both the direction and magnitude of the effect are consistent and not driven by any single study. Figure 6 presents the results of the studies included in the context of depression symptoms.

Negative SMD values indicate reductions in depressive symptoms favoring probiotic supplementation. The green markers denote studies in which probiotic supplementation produced a beneficial or clinically meaningful reduction in depressive symptoms; the red markers denote studies in which the effect either (a) did not reach statistical significance or (b) yielded confidence intervals crossing the null value, indicating no reliable evidence of a treatment effect.

#### 3.3.5. Effects of Probiotic Use on Symptoms of Anxiety

As with the procedures applied to the analysis of probiotic effects in patients with MDD, all meta-analyses reporting anxiety outcomes in terms of mean differences were converted to SMD to ensure full comparability and scale-independent interpretation across studies. This standardization allowed the anxiety-related effect sizes to be compared consistently, regardless of the instrument originally used to measure symptom severity.

**Figure 7:**
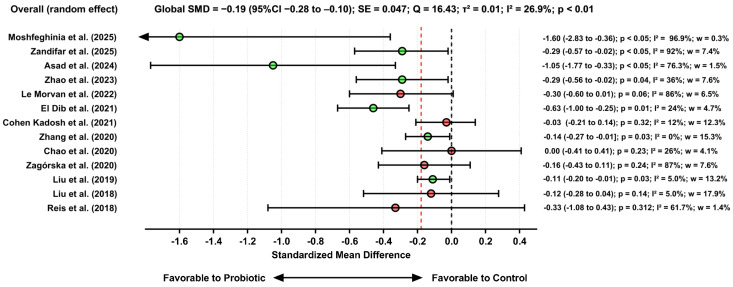
Effects of probiotic supplementation on anxiety symptoms. Individual study estimates are represented by circles, with horizontal lines indicating their respective confidence intervals; the size of each marker reflects the study’s relative weight in the random-effects model. Studies on probiotics and anxiety include: Reis et al. [31]; Liu et al. [30]; Liu et al. [32]; Zagórska et al. [35]; Chao et al. [36]; Zhang et al. [37]; Cohen Kadosh et al. [41]; El Dib et al. [42]; Le Morvan et al. [16]; Zhao et al. [45]; Asad et al. [48]; Zandifar et al. [51]; Moshfeghinia et al. [53].

The meta-analysis based on 13 effect estimates showed that probiotics significantly reduced anxiety symptoms (SMD = −0.19; 95% CI: −0.28 to −0.10; *p* < 0.01). Heterogeneity was moderate (I^2^ = 26.9%), indicating substantial variability among studies. Sensitivity analyses demonstrated that both the magnitude and direction of the effect remained stable after the sequential removal of influential studies. Figure 7 reports the results for the anxiety scores.

Forest plot showing standardized mean differences (SMD) and 95% confidence intervals for anxiety outcomes derived from subgroup analyses or primary estimates from individual meta-analyses. Negative SMD values indicate reductions in anxiety symptoms favoring probiotics.

The sensitivity analysis demonstrated a significant degree of stability in the estimates. Sequential removal of each individual study showed that the direction of the effect remained unchanged. Removing the study with the largest absolute effect size (Moshfeghinia et al. [53]), which reported an SMD of −1.60 [95% CI: −2.83 to −0.36], resulted in a modest reduction in the overall effect (recalculated SMD = −0.28; 95% CI: −0.39 to −0.17), while maintaining statistical significance. Additional exclusion of other studies with extreme effects, such as Asad et al. [48] (SMD = −1.05; 95% CI: −1.77 to −0.33) and El Dib et al. [42] (SMD = −0.63 [95% CI: −1.00 to −0.25], yielded pooled estimates that remained within a consistent range (SMD between −0.24 and −0.31, with 95% CIs still below zero), reinforcing that the magnitude of the effect was not driven by any single outlier study.

#### 3.3.6. Effects of Prebiotic Use on Symptoms of Depression

It is important to highlight that Zandifar et al. [51] did not stratify their analyses by type of intervention (probiotics, prebiotics, or synbiotics). This lack of segmentation introduces contamination among treatment effects, rendering the results unsuitable for deriving conclusions specific to prebiotic interventions. Therefore, the article was removed from the analysis.

**Figure 8:**
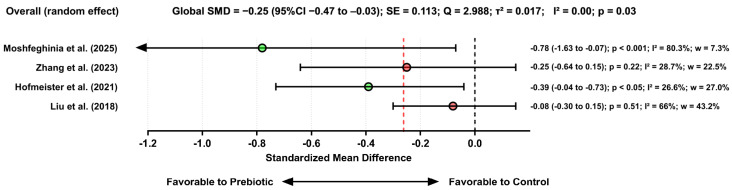
Effects of prebiotic supplementation on depressive symptoms. Individual study estimates are represented by circles, with horizontal lines indicating their respective confidence intervals; the size of each marker reflects the study’s relative weight in the random-effects model. Studies on prebiotics and depression include: Liu et al. [30]; Hofmeister et al. [39]; Zhang et al. [37]; Moshfeghinia et al. [53].

The quantitative synthesis of the four studies evaluating the effects of prebiotics on depressive symptomatology demonstrated a small but statistically significant reduction in depression scores among participants receiving prebiotic supplementation. Using a random-effects model, the pooled effect estimate indicated a global SMD = −0.25 (95% CI: −0.47 to −0.03; *p* = 0.03; τ^2^ = 0.017), suggesting that prebiotics confer a modest improvement in depressive symptoms when compared with control conditions. Figure 8 reports the effects of prebiotics on depression scores.

To analyze the potential influence of outliers on the effects of prebiotics on depression scores, a sensitivity analysis was conducted to determine whether the overall effect estimate was disproportionately influenced by any individual study. Given that Moshfeghinia et al. [53] reported the largest effect size among the included trials (SMD = −0.78; 95%CI −1.63 to −0.07), this study was systematically removed to assess its impact on the pooled effect. When excluding Moshfeghinia et al. [53], the remaining studies, Zhang et al. [53] (SMD = −0.25; 95%CI −0.64 to 0.15), Hofmeister et al. [39] (SMD = −0.39; 95%CI −0.73 to −0.04), and Liu et al. [32] (SMD = −0.08; 95%CI −0.30 to 0.15), were recombined using a random-effects model.

The recalculated pooled effect shifted to −0.19 [95%CI −0.24 to −0.03], Q = 1.936; SE = 0.095, not crossing the null value and, therefore, becoming statistically significant (*p* < 0.05). This finding indicates that the significant overall effect originally observed was partially dependent on the contribution of the Moshfeghinia study, which presented a notably larger effect than the remaining body of evidence.

#### 3.3.7. Effects of Prebiotic Use on Symptoms of Anxiety

**Figure 9:**
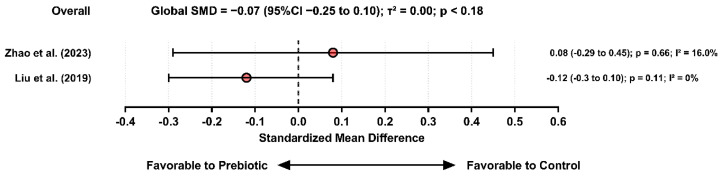
Effects of prebiotic supplementation on anxiety symptoms. Individual study estimates are represented by circles, with horizontal lines indicating their respective confidence intervals; the size of each marker reflects the study’s relative weight in the random-effects model. Studies on prebiotics and anxiety include: Liu et al. [32]; Zhao et al. [45].

Only two meta-analytic estimates evaluating the effects of prebiotic supplementation on anxiety outcomes were included in the quantitative synthesis. Individually, the studies demonstrated small and statistically non-significant effects. Zhao et al. [45] reported a SMD of 0.08 [95% CI: −0.29 to 0.45], *p* = 0.66; I^2^ = 16%, indicating no detectable anxiolytic benefit of prebiotics. Similarly, Liu et al. [32] found an SMD of −0.12 (95% CI: −0.30 to 0.10; *p* = 0.11; I^2^ = 0%), also failing to demonstrate meaningful reductions in anxiety symptoms. When combined under a random-effects model, the pooled effect demonstrated a non-significant trend favoring prebiotics. The small magnitude and imprecision of the effect highlight the need for more robust, well-designed clinical trials specifically targeting anxiety-related outcomes. Figure 9 presents the studies included for analysis.

### 3.4. Risk-of-Bias Assessments (AMSTAR 2)

The methodological appraisal using the AMSTAR 2 framework revealed substantial variability in the confidence attributed to the included systematic reviews and meta-analyses, highlighting marked heterogeneity in their overall robustness. Among the 31 reviews assessed, only 12 (38.7%) achieved a rating of high confidence [16,17,27,28,30,31,33,39,44,48,53,54] characterized by the absence of critical flaws and by strong adherence to core methodological standards, particularly comprehensive search strategies, rigorous risk-of-bias assessments, transparent synthesis protocols, and registration in the PROSPERO database or similar. We highlight that, despite the generally high adherence to methodological quality criteria and the absence of critical flaws, some reviews exhibited shortcomings in adequately reporting specific AMSTAR 2 checklist items. Consequently, their overall compliance did not reach 100% [17,28,31,33].

A smaller subset, four reviews (12.9%), was classified as moderate confidence, typically due to the presence of isolated non-critical weaknesses, yet maintaining an overall coherent and well-structured methodological approach. Although none of these reviews exhibited critical flaws according to the AMSTAR 2 criteria, several demonstrated non-critical weaknesses that limited the completeness of their methodological reporting. For example, Zhang et al. [37] did not adequately describe the procedures for duplicate study selection and duplicate data extraction, which are essential for minimizing operational errors and bias. Similarly, Zhao et al. [45] also failed to clearly report duplicate study selection, compromising methodological transparency; El Dib et al. [42] presented gaps in the detailed description of included studies. In addition, Zhao et al. [50] did not explicitly address the assessment of heterogeneity or the potential sources of variability.

Strikingly, eleven studies were rated as low confidence (35.4%), reflecting recurrent deficits across multiple AMSTAR2-critical domains [32,34,35,36,40,41,43,46,47,49,52]. The most frequent shortcomings included lack of protocol registration (Item 2), inadequate or poorly justified search strategies (Item 4), failure to account for risk of bias in primary studies when interpreting findings (Item 13), and omission of publication bias assessment (Item 15). These recurrent methodological gaps suggest that substantial portions of available syntheses provide limited reliability for decision-making, despite often reporting statistically significant results.

**Figure 10:**
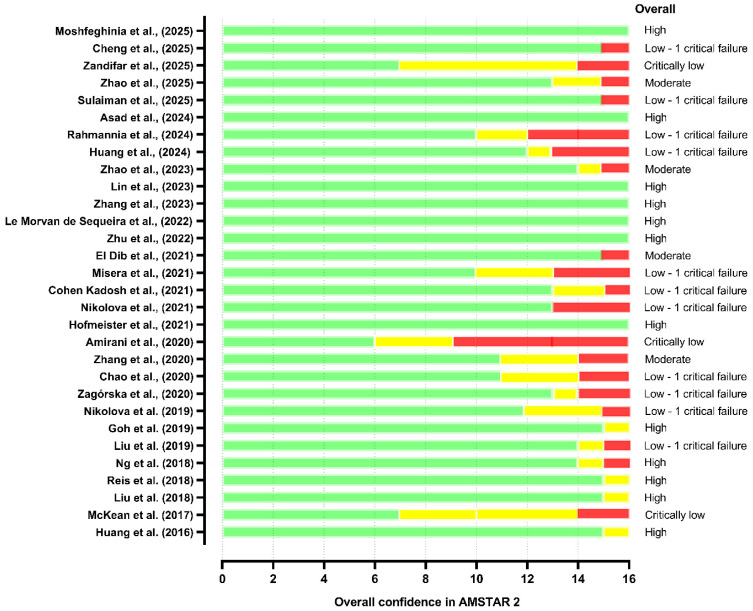
Summary of overall confidence in the results of each meta-analysis. The AMSTAR 2 analysis included the following studies: Huang et al. [28]; McKean et al. [29]; Reis et al. [31]; Ng et al. [17]; Liu et al. [30]; Liu et al. [32]; Goh et al. [33]; Nikolova et al. [34]; Zagórska et al. [35]; Chao et al. [36]; Zhang et al. [37]; Amirani et al. [38]; Hofmeister et al. [39]; Nikolova et al. [40]; Cohen Kadosh et al. [41]; El Dib et al. [42]; Misera et al. [43]; Zhu et al. [54]; Le Morvan et al. [16]; Zhang et al. [27]; Lin et al. [44]; Zhao et al. [45]; Huang et al. [46]; Rahmannia et al. [47]; Asad et al. [48]; Sulaiman et al. [49]; Zhao et al. [50]; Zandifar et al. [51]; Cheng et al. [52]; Moshfeghinia et al. [53].

Finally, four reviews (12.9%) scored critically low confidence, primarily because they contain multiple critical flaws [29,38,51,58]. These studies, in particular, exhibit failures in fundamental reporting processes, such as the following: (a) disclosure of funding sources and conflicts of interest; (b) insufficient or low-quality reporting of risk of bias; (c) inadequate assessment of heterogeneity; and (d) lack of protocol registration in the PROSPERO database. This pattern not only reflects variability in methodological maturity among research groups and publication periods but also emphasizes the importance of cautious interpretation when integrating findings from reviews with suboptimal quality. Table S4 details the items classified for the AMSTAR 2 scale. Figure 10 presents the judgment criteria for the AMSTAR2 checklist.

The overall confidence in the results of each systematic review was rated according to AMSTAR 2 guidelines, based on the presence and number of critical and non-critical weaknesses. Critical domains include the following: (2) protocol registration, (4) adequacy of the search strategy, (7) listing of excluded studies with justification, (9) risk of bias assessment of included studies, (11) appropriateness of meta-analytic methods, (13) consideration of risk of bias when interpreting results, and (15) assessment of publication bias. High confidence: No critical flaws and at most one non-critical weakness; moderate confidence: No critical flaws but more than one non-critical weakness; low confidence: One critical flaw with or without non-critical weaknesses; and critically low confidence: Two or more critical flaws with or without other weaknesses. The green bar represents items fully met according to the AMSTAR-2 criteria. The yellow bar indicates items that were only partially met or presented with limited methodological detail. The red bar denotes items that were not met and correspond to potential critical and/or non-critical weaknesses in the methodological quality of the included reviews.

### 3.5. Heterogeneity of the Included Studies

The assessment of heterogeneity among the primary studies within each meta-analysis revealed substantial variability, with I^2^ values ranging from minimal (0.8%) to extremely high (99.7%). According to the interpretation framework recommended by the Cochrane Handbook, only five meta-analyses (16.1%) demonstrated low heterogeneity (I^2^ ≤ 25%), indicating a high degree of consistency across their primary studies [17,32,41,42], whose findings are less influenced by methodological or clinical diversity.

Moderate heterogeneity (I^2^ = 25% to 50%) was observed in five studies (16.1%), including [27,28,36]. This level of variability is generally acceptable and often reflects moderate differences in sample characteristics, interventions, or outcome measures. In contrast, the largest proportion of meta-analyses fell within the category of substantial heterogeneity (I^2^ = 50% to 75%), comprising nine studies (29.0%), such as [30,31,43,46,49,58]. This degree of inconsistency suggests that moderate-to-marked differences in methods, populations, or analytical approaches contributed significantly to the observed variance.

Finally, a considerable number of meta-analyses (twelve studies; 38.7%) exhibited very high heterogeneity (I^2^ > 75%), indicating pronounced disagreement among the included primary studies. This group includes [16,33,34,35,38,48,53], with values exceeding 75% suggesting that methodological, clinical, or statistical factors strongly influence the consistency of aggregated effects.

## 4. Discussion

This umbrella review synthesized and critically appraised 30 systematic reviews and meta-analyses evaluating the effects of psychobiotic interventions on depressive and anxiety symptoms in adults with a confirmed diagnosis or with symptoms consistent with the disorders. Overall, the evidence indicates that probiotics demonstrate moderate and consistent beneficial effects on depressive symptoms, whereas findings for anxiety show only minor effects. Additionally, in the context of prebiotics, only a significant, but small, effect was observed for symptoms of depression, whereas for anxiety, the body of evidence available in the literature is poor. Supplementation with synbiotics remains limited. These results align with previous evidence suggesting that gut–brain modulation may influence affective regulation [61].

The anxiolytic and antidepressant potential of psychobiotics is supported by multiple interrelated biological mechanisms centered on neurotransmitter modulation, gut–brain axis signaling [61], immune regulation, and maintenance of intestinal homeostasis [8]. By modulating these interconnected pathways, psychobiotics create a physiological environment conducive to improvements in mental health, particularly in anxiety and depression, consistent with observations in our study. A key mechanism involves the microbial stimulation of neurotransmitter synthesis in the gut, which functions as a major neurochemical hub [61]. Certain *Lactobacillus* species can produce acetylcholine, thereby influencing both peripheral and central cholinergic pathways linked to mood regulation and gastrointestinal function [62,63]. Similarly, several *Bifidobacterium* and *Lactobacillus* strains are capable of producing gamma-aminobutyric acid (GABA), the principal inhibitory neurotransmitter in the CNS [62]. Microbially derived GABA may modulate stress reactivity through gut–brain communication, contributing to reductions in anxiety-like behaviors and improved mood [62,63,64].

However, despite the relevant results, our findings reveal substantial variability in the estimated effects, probably resulting from the different strains employed, the administered doses, the combinations of microorganisms (single-strain vs. multi-strain), and the type of formulation used (fermented foods, capsules). This heterogeneity hinders direct comparison of results and prevents the identification of which specific psychobiotic may be most effective for a given condition. At present, no consensus exists regarding the optimal strain or strain combination, nor the ideal dosage or duration required to exert antidepressant or, principally, anxiolytic effects. This variability contributes to inconsistent findings; for example, some meta-analyses have detected significant improvements on scales such as the BDI (SMD = −2.69 [95%CI: −4.22 to −1.16]; *p* < 0.001), but not on the HAM-D (SMD = −1.40 [95%CI: −3.29 to 0.48]; *p* = 0.14) (Rahmannia et al. [47]). The same occurs when we observe the standardized effect sizes for BDI and the MADRS, which are reasonably different, suggesting that methodological differences may influence overall conclusions [47]. In our study, we observed that the main scale used among primary studies was the BDI, which we suggest is the most consistent tool. Some readers may even caution that, given the plethora of different probiotic, prebiotic, and synbiotic approaches available, it could be seen as daring to pool the analysis of the effect of such diverse treatments. Moreover, both depression and anxiety are symptoms of different underlying etiologies: Patients may respond differentially to a specific treatment in the context of terminal cancer, PTSD, postpartum depression, Parkinson’s disease, or dementia. Our study corroborates that the gut is an auspicious target to augment patient health that goes far beyond its immediate effect, via mechanisms that we are barely starting to comprehend. So far, it is not clear how the choice of a particular biotic can be personalized to be best suited for specific patient needs. We anticipate that further exploration into that realm will be commensurate with the investment.

We also observed that many trials tested probiotics in healthy or subclinical volunteers, focusing on prevention or general well-being, which frequently yielded no or attenuated effects. This mismatch limited the extrapolation of findings to individuals with diagnosed depressive or anxiety disorders, who constitute the primary clinical population of interest. Reviews such as Ng et al. [17] and Reis et al. [31] highlighted this gap, noting that most available RCTs at the time did not involve participants with established mental disorders, mostly anxiety disorders. What we aimed to achieve in this paper was precisely to focus on diagnosed and subclinical populations, providing more specific insights by integrating evidence from different studies. Thus, for depressive symptoms, we observed a significant estimated effect SMD = −0.50; 95% CI = −0.58 to −0.42, *p* = 0.0001), providing support for an additional viable therapeutic strategy, particularly for patients who are refractory to conventional treatments.

Smith et al. [65] and Misera et al. [43] reported differences in clinical outcomes linked to changes in immunological and inflammatory biomarkers (e.g., hs-CRP, IL-6, and TNF-α) and oxidative markers, which collectively could explain the reduction in depressive symptoms. Furthermore, Nikolova et al. [40] reported a significant reduction in CRP and an increase in brain-derived neurotrophic factor (BDNF) among participants receiving probiotics as an adjuvant intervention, suggesting immunomodulatory and neuroplastic mechanisms as potential pathways of action. The increase in BDNF derived from probiotic supplementation is observed mainly in patients with depression and neurological disorders, and a mixture of *Lactobacillus* and *Bifidobacterium* appears to show greater efficacy in reducing depressive scores [20]. These findings suggest a potential anti-inflammatory and neuroprotective mechanism mediated through the gut–brain axis.

In other hands, our findings in the context of probiotic supplementation to mitigate the effects of anxiety were also significant (SMD = −0.19; 95% CI: −0.28 to −0.10; *p* < 0.01), but, it is worth noting that, elevated levels of anxiety, i.e., an exaggerated, transient, and functional response, do not constitute an anxiety disorder, which is a sustained neurobiological dysfunction accompanied by functional impairment [66,67]. However, the primary study led by Eskandarzadeh et al. [68] reported a greater reduction in anxiety symptoms among patients with generalized anxiety disorder who received a probiotic plus sertraline (HAM-A: −11.84 ± 8.08) compared with those who received placebo plus sertraline (HAM-A: −8.52 ± 6.85; *p* = 0.003). Thus, although our findings indicate overall limited effects, this result suggests that probiotics may hold potential as an adjuvant intervention for anxiety disorders. For now, we may regard these findings as a promising indicator for future experiments targeting anxiety disorders.

Although prebiotics represent a biologically plausible strategy for modulating mood, primarily through fermentation-derived production of short-chain fatty acids (SCFAs), enhancement of microbial diversity, and regulation of gut–immune pathways, the current body of evidence remains limited and heterogeneous [69,70]. In the meta-analyses included in this umbrella review, the effects of prebiotic supplementation on depressive or anxiety symptoms were generally small, SMD = −0.25 (95% CI: −0.47 to −0.03; *p* = 0.03; τ^2^ = 0.017), or non-significant, SMD = −0.07 [95% CI: −0.25 to 0.10], *p* = 0.18, (in the case of anxiety symptoms). These findings are consistent with previous analyses suggesting that the psychotropic potential of prebiotics is markedly less important than that observed for probiotics.

Prebiotics such as GOS and FOS, most commonly seen in meta-analyses, selectively stimulate beneficial taxa capable of producing SCFAs-butyrate, whose downstream actions include modulation of inflammatory signaling, enhancement of intestinal barrier integrity, and regulation of serotonergic pathways [71]. However, clinical translation of these mechanisms has been inconsistent, and the efficacy may depend on the host’s baseline microbiota composition, which varies widely across populations and tends to be less responsive to isolated fiber supplementation compared with multi-strain probiotic formulas [72]. This contextual dependency is reflected in trials of GOS that reported improvements only among individuals with high baseline anxiety, but not in broader samples, suggesting a potential ceiling effect or subgroup-specific responsiveness.

So, current evidence indicates that prebiotics alone exert limited and inconsistent effects on mental health outcomes. Their therapeutic relevance may be better understood in the context of synbiotic formulations, which demonstrated more promising, albeit still limited, results for depressive symptoms (Moshfeghinia et al. [53]: SMD = −1.25 [95% CI −1.91 to −0.58]; *p* < 0.001) and anxiety (Zhao et al. [45]: SMD = −0.71 [95% CI −1.04 to −0.38]; *p* < 0.01). These findings suggest that the synergistic interaction between prebiotics and probiotics may enhance their psychobiotic potential, rather than prebiotics functioning as effective stand-alone interventions.

It is important to highlight that specific population subgroups remain markedly underexplored in current systematic reviews with meta-analyses. Older adults, particularly those over 60 years of age, frequently exhibit age-related alterations in gut microbiota composition, compounded by multimorbidity and polypharmacy. These factors may influence their responsiveness to probiotic supplementation; however, trials conducted in elderly populations are scarce. In the limited evidence available, Huang et al. [28] reported no significant effect of probiotics among participants older than 65 years (SMD = −0.18 [95%CI: −0.47 to 0.11]; *p* = 0.22), in contrast to younger adults, which is likely attributable to the small number of older individuals included. However, similar findings were observed in Zhu et al. [54] and Cheng et al. [52], which also failed to demonstrate significant probiotic effects compared with placebo or control groups (SMD = −0.13 [95%CI: −0.31 to −0.04], *p* > 0.05 and SMD = −0.22 [95%CI: −0.57 to 0.13], *p* = 0.12, respectively). These results highlight a persistent evidence gap concerning psychobiotic efficacy in older adults, underscoring the need for targeted, age-specific clinical research in this demographic.

### 4.1. Interaction Between Antidepressants and Probiotics/Prebiotics

It is important to contextualize these findings within the broader pharmacological landscape of depression treatment, particularly regarding the widespread use of antidepressants such as selective serotonin reuptake inhibitors (SSRIs). SSRIs remain first-line therapy for major depressive disorder; however, their clinical effectiveness is frequently limited by delayed onset of action, incomplete remission rates, substantial interindividual variability in response, and the occurrence of adverse effects, notably gastrointestinal symptoms [73,74].

Evidence indicates that antidepressants themselves may interact bidirectionally with the gut microbiota [74]. Several SSRIs exhibit intrinsic antimicrobial activity capable of altering microbial composition and diversity, which may influence both therapeutic efficacy and tolerability [2,62,64,72,75]. These microbiota alterations may partially explain antidepressant-induced gastrointestinal side effects and, potentially, variability in clinical response [76]. Conversely, baseline dysbiosis has been associated with altered antidepressant metabolism and reduced treatment responsiveness, suggesting that the gut microbiome may act as a moderator of pharmacological efficacy [7,76,77].

Probiotics and prebiotics may complement antidepressant therapy through convergent modulation of the microbiota–gut–brain axis [1,10,23,64,72,78,79]. Proposed pathways include increased production of short-chain fatty acids, regulation of inflammatory and immune signaling, modulation of tryptophan metabolism and serotonergic neurotransmission, reinforcement of intestinal barrier integrity, and attenuation of HPA axis hyperactivity [62,80]. Several of these mechanisms overlap with biological targets indirectly influenced by antidepressant drugs, supporting a biologically plausible rationale for combined or adjunctive approaches [62].

Confirming this, a recent high-quality systematic review and network meta-analysis by Zhao et al. [50] directly compared microbiota-targeted therapies with antidepressants in adults with MDD. Among 42 RCTs, probiotics demonstrated significant reductions in depressive symptom severity compared with placebo and were non-inferior to multiple antidepressants, including venlafaxine, vortioxetine, duloxetine, and citalopram. Notably, probiotics ranked second only to escitalopram in the treatment hierarchy and showed comparable tolerability to antidepressants when administered for ≥8 weeks. Moreover, probiotic interventions used as adjuncts to antidepressant therapy were superior to several pharmacological agents alone.

So, preclinical and clinical studies provide support for this interaction, demonstrating that specific probiotic strains can exert antidepressant-like effects and possibly modulate stress-related neuroendocrine and inflammatory responses, sometimes comparable to conventional antidepressants [1,28,38,41,46,48,52,60,68,81].

### 4.2. Strain-Specific Mechanistic Pathways Linking Lactobacillus and Bifidobacterium

It is important to highlight that a critical characteristic of the trials included in this review is the predominance of Lactobacillus and *Bifidobacterium* strains, administered either as single strains or in multispecies formulations. This observation is relevant because the biological effects attributed to probiotics are not uniform but rather depend on strain-specific and functional properties. Psychobiotic effects arise from specific microbial capacities related to metabolite production, immune modulation, and host–microbe signaling, rather than from probiotic exposure per se [6,49,62,82].

Experimental evidence provides robust support for neural communication as one of the pathways linking specific *Lactobacillus* strains to the regulation of stress and affective states. Chronic administration of *Lactobacillus rhamnosus* (JB-1) has been shown to induce region-specific alterations in GABA receptor expression and to attenuate stress-related behaviors; notably, these effects are abolished following vagotomy, identifying the vagus nerve as a critical conduit for gut–brain signaling [81]. Cryan et al. [62] position this vagal pathway as one of the most biologically coherent routes of gut–brain communication. However, despite its strong biological plausibility, direct confirmation of vagal mediation in the improvement of depressive and anxiolytic symptoms in humans remains limited, as few randomized controlled trials have simultaneously assessed vagal biomarkers.

In addition, the low-grade systemic inflammation commonly observed in patients with depressive disorders constitutes a biologically plausible target for microbiota-mediated interventions. Both *Lactobacillus* and *Bifidobacterium* strains have demonstrated anti-inflammatory properties, including reductions in pro-inflammatory cytokines and markers of oxidative stress. Clinical trials have reported concurrent improvements in depressive symptom scores alongside reductions in hs-CRP, IL-6 expression, and oxidative stress indices following probiotic supplementation [1,38]. Sulaiman et al. [49] identify immune modulation as one of the most consistently supported mechanisms across human studies, suggesting that attenuation of peripheral inflammation may indirectly influence neuroinflammatory processes and HPA activity. Among the proposed pathways, immune–brain signaling currently represents one of the strongest translational links between probiotic exposure and reductions in depressive symptoms.

Another frequently cited mechanism involves the regulation of tryptophan availability. Certain *Bifidobacterium* strains have been associated with alterations in tryptophan metabolism and increased peripheral serotonin availability in clinical trials [83]. This pathway provides a coherent intersection between immune activation, microbial function, and neurotransmission. However, it is now well-recognized that depression and its comorbidities do not depend exclusively on monoaminergic pathways, and, therefore, this mechanism should be interpreted with caution [61,84].

Emerging neuroimaging data further suggest that probiotic interventions may influence brain structure and functional connectivity within frontolimbic circuits implicated in mood regulation. Changes in hippocampal activation patterns, fractional anisotropy, and gray matter volume have been reported in small clinical samples following supplementation with multispecies probiotics [85,86]. Although these findings are intriguing, further studies are required to confirm and extend these observations.

### 4.3. Methodological Caveats

During the process of collecting and synthesizing the various meta-analyses, we identified potential sources of confusion, including the directionality of reported outcomes in some studies. When evaluating effects based on psychometric instruments such as the BDI, HAM-D, or DASS, one expects results to be expressed as reductions in symptom scores, given that lower scores correspond to clinical improvement. Consequently, when reporting effect estimates, the direction of the effect should mathematically be negative (post-intervention values must be lower than baseline for the intervention to be interpreted as beneficial).

However, the meta-analyses by Nikolova et al. [34], Nikolova et al. [40], and Hofmeister et al. [39] reported clusters of positive SMDs, resulting in an overall pooled effect that was also positive (SMD = 0.86 [95%CI: −0.57 to 2.17], *p* > 0.05; SMD = 0.58 [95%CI: 0.19 to 0.97], *p* < 0.05; and SMD = 0.78 [95%CI: 0.19 to 1.37], *p* < 0.05, respectively). When these data were synthesized alongside the remaining literature, we observed an irregular pattern: the direction of the effect in these three studies favored the control or placebo group, despite the authors’ conclusions being favorable to supplementation. Accordingly, it was necessary to adjust the direction of these effect sizes to ensure consistent alignment and appropriate aggregation within our analysis.

Additionally, the meta-analysis by Nikolova et al. [34] appeared to contain several methodological inconsistencies. For instance, Kazemi et al. [60] were included alongside Akkasheh et al. [1] and Romijn et al. [79]. The estimated effect size for Kazemi et al. [60] was strikingly large (SMD = 1.99 [95% CI: 1.43 to 2.55]), which would only be plausible if the authors had reported mean differences rather than standardized mean differences, which they did not. When cross-referencing these values with more recent meta-analyses published between 2020 and 2025, such as Misera et al. [43], the same trial yields an SMD of −0.39 [95% CI: −0.85 to 0.07], i.e., a markedly different effect estimate. This discrepancy strongly suggests a potential error in the earlier review, which, in turn, compromises the validity of its conclusions.

Finally, the inclusion of multiple interventions, such as probiotics, prebiotics, and synbiotics, within the same dataset, without appropriate subgroup analyses or with missing subgroup information, compromises the interpretability of findings in subsequent research. Zandifar et al. [51], for example, examined the effects of prebiotics and probiotics on depression, anxiety, and cognition; however, the authors only reported a combined effect estimate, pooling prebiotics, probiotics, and synbiotics. Although they stated that subgroup analyses had been performed, these results were not adequately reported (there is no Supplementary Materials section). Other reviews similarly lack essential information when dealing with multiple intervention types, particularly in the context of synbiotic supplementation, further complicating the synthesis and comparison of effects across studies. In our case, this issue eliminated the possibility of adequately processing the data to estimate the effects of synbiotics.

### 4.4. Analysis of Methodological Quality

The methodological assessment conducted in this umbrella review revealed a consistent pattern of substantial weaknesses among most of the included systematic reviews and meta-analyses, which is in line with the limitations previously identified by Morán et al. [23]. Using the AMSTAR 2 instrument, we found that the majority of reviews were classified as low or critically low in overall confidence. This distribution closely mirrors the findings of Morán et al. [23], who also reported a predominance of low-confidence ratings, highlighting Hofmeister et al. [39] as the only review meeting the high-quality standard.

One of the most frequent shortcomings was the absence of a pre-registered protocol (Item 2), which compromises transparency and increases the risk of post hoc decision-making. Recurrent issues also included inadequate reporting of funding sources for the included studies (Item 10) and the absence or insufficiency of publication bias assessments (Item 15). Additionally, as noted by Morán et al. [23], only a portion of the reviews appropriately incorporated risk-of-bias evaluations into the interpretation of their findings (Item 13). Even when such assessments were performed, their implications were seldom discussed with the necessary depth.

Another recurring problem was the high level of statistical heterogeneity, with I^2^ values frequently exceeding 70%. Although some reviews conducted subgroup or sensitivity analyses to mitigate this issue, substantial heterogeneity persisted. Key contributors included notable differences in population characteristics (healthy individuals vs. clinically diagnosed patients), baseline symptom severity, intervention dosage and duration, and variability in the psychometric instruments used to assess outcomes.

So, as indicated by the AMSTAR 2 assessment, several of the included studies exhibited critical or high-risk methodological weaknesses, which constrain the confidence that can be placed in pooled estimates. These limitations are largely inherent to the existing evidence base and cannot be corrected retrospectively within the present synthesis. Consequently, while observed effects may indicate the potential benefits of psychobiotic interventions, the findings should be interpreted with caution and should not be considered definitive evidence of efficacy.

### 4.5. Future Directions and Limitations

A major source of heterogeneity among the included studies likely arises from the substantial variability in probiotic and prebiotic formulations rather than from differences in outcome assessment alone. Across primary trials, interventions differed markedly with respect to microbial strains, combinations of species, dosages, intervention duration, and delivery formats. Such compositional heterogeneity may exert a stronger influence on observed effects than the choice of psychometric instruments used to assess depressive or anxiety symptoms.

Although some recent studies have attempted to address this issue by employing standardized or well-characterized strains, the majority of available trials continue to use heterogeneous formulations, limiting the ability to attribute effects to specific microbial profiles. The present review did not perform strain-specific or dose–response analyses, which represents an important limitation and reflects the current constraints of the evidence base. As illustrated in Table 1, the wide diversity of probiotic compositions precludes firm conclusions regarding the relative efficacy of individual strains or combinations. Future research should prioritize standardized formulations and strain-specific designs to reduce heterogeneity and enable more precise mechanistic and clinical inferences.

Additionally, while the adoption of a single universal outcome measure could, in theory, enhance comparability, such an approach is unlikely to be feasible or desirable given differences in study objectives, populations, symptom severity, and clinical versus subclinical contexts. From a practical and methodological standpoint, a more realistic solution lies in the standardization of analytical frameworks rather than in the exclusive use of a single instrument. In this review, comparability was addressed by prioritizing effect estimates derived from clinically relevant subgroups when available and by selecting SMDs based on instruments with stronger psychometric support and greater prevalence among trials. Global SMDs were retained when populations were homogeneous or when further stratification would compromise statistical robustness.

Future research should move beyond highly generalized analyses that pool heterogeneous populations, symptom profiles, and probiotic formulations, as such approaches may obscure strain-specific effects and attenuate clinically meaningful signals. Accumulating evidence suggests that probiotic effects on mental health outcomes are not uniform [47,64,69,82,83], but instead depend on clearly defined strain characteristics and well-characterized participant profiles. Accordingly, future trials should prioritize more phenotypically and biologically specific study designs, including the evaluation of single strains, that are well-justified.

In parallel, greater emphasis should be placed on recruiting clinically defined populations, such as individuals with established diagnoses of depressive or anxiety disorders, or on clearly stratifying participants by symptom severity and baseline microbiota characteristics. It is worth highlighting that only a limited number of existing studies have adopted such stringent designs, focusing on specific strains or clinically diagnosed populations (observed in more recent studies) [47,64,69,82,83], and yet these studies tend to provide clearer and more interpretable effect estimates.

Finally, despite growing interest in microbiota-targeted interventions, the current evidence base remains heavily skewed toward prebiotic formulations, limiting the ability to differentiate the effects on depressive and anxiety symptoms. Future progress in this field will require methodologically rigorous primary studies specifically designed to evaluate prebiotics and synbiotics, with clearly defined compositions.

## 5. Conclusions

This umbrella review demonstrates that psychobiotic interventions are more consistently associated with reductions in depressive symptoms than in anxiety symptoms, for which findings remain heterogeneous and less stable. Probiotics emerged as the most frequently investigated intervention, demonstrating modest but generally favorable effects on depressive symptoms. Evidence for prebiotics and, particularly, synbiotics was limited in both contexts, with only a small number of reviews providing extractable estimates, and several failing to isolate intervention types or to stratify different formulations.

Despite the substantial growth in the number of meta-analyses published, the overall certainty of the evidence remains constrained by methodological limitations, inconsistent reporting, and substantial heterogeneity among reviews. Consistent with this, most reviews demonstrated low-to-moderate confidence according to the AMSTAR 2 framework, primarily due to critical weaknesses in protocol registration, search completeness, selection bias, and integration of risk-of-bias assessments.

Citation-based mapping and timeline analyses further revealed an uneven accumulation of evidence, characterized by clusters of redundant reviews and limited conceptual connectivity across the field.

## Figures and Tables

**Table 1:** Characteristics of systematic reviews and meta-analyses included in the umbrella review.

Author/Year (Type; n Total)	Population (n, Age, Condition)	Intervention (Probiotic Species/Duration)	Main Outcome and Direction of Change
Huang et al. [28] Meta-analysis (5 RCT; n = 365)	n = 183 EXP, n = 182 PLA. Samples: healthy adults (n = 3); Stressed Workers (n = 1); Patients with MDD (n = 1). Age range: 20–65 years.	Probiotics: *L. casei*, *L. acidophilus*, *L. rhamnosus*, *L. bulgaricus*, *L. pentosus*, *L. helveticus*, *L. brevis*, *L. salivarius*, *L. lactis*, *B. breve*, *B. longum*, *B. bifidum*, *B. lactis*, *S. thermophilus*., sachets, yogurt; Duration: 4–20 weeks.	**Global: SMD = −0.30 [95%CI −0.51 to −0.09]; *p* = 0.005.** Subgroups: **<60 years: SMD = −0.43 [95% CI −0.72 to −0.13]; *p* = 0.005.** ≥65 years: NS; SMD = −0.18; [95%CI −0.47 to 0.11]; *p* = 0.22. **Healthy (n = 4): SMD = −0.25 [95%CI −0.47 to −0.03]; *p* = 0.03** **MDD (n = 1): SMD = −0.73 [95%CI −1.37 to −0.09]; *p* = 0.03.**
Liu et al. [30] Meta-analysis (12 RCT; n = 1551)	n = 871 EXP, n = 680 PLA. Healthy participants (n = 4) Irritable eye syndrome (n = 1) Patients with MDD (n = 1) Patients with IBS (n = 6), Age ranged from 19.7 to 72.6 years; most participants were women.	Probiotics: *L. helveticus*, *L. rhamnosus*, *L. acidophilus*, *L. casei Shirota*, *L. plantarum 299 v*, *L. reuteri*, *L. delbrueckii bulgaricus*, *L. lactis*, *B. longum*, *B. breve*, *B. bifidum*, *B. lactis*, *B. animalis*, *Pediococcus acidilactici*, *S. thermophilus*; Forms: capsules, sachets, fermented milk/yogurt Duration: 4–24 weeks.	Global: SMD = −0.12 [95% CI −0.28 to 0.04]; *p* = 0.14; I^2^ = 51% → NS. Healthy (n = 4): SMD = −0.18 [95% CI −0.41 to 0.05]; *p* > 0.05 Unhealthy (n = 8): SMD = −0.09 [95% CI −0.30 to 0.13]; *p* > 0.05 IBS (n = 6): SMD = −0.10 [95% CI −0.36 to 0.16]; *p* > 0.05 Non-IBS (n = 6): SMD = −0.14 [95% CI −0.31 to 0.03]; *p* > 0.05 ≤8 weeks (n = 8): SMD = −0.11 [95% CI −0.34 to 0.12]; *p* > 0.05 >8 weeks (n = 4): SMD = −0.14 [95% CI −0.37 to 0.09]; *p* > 0.05 Multi-strain (n = 7): SMD = −0.05 [95% CI −0.26 to 0.17]; *p* > 0.05 Single-strain (n = 5): SMD = −0.21 [95% CI −0.44 to 0.02]; *p* > 0.05
Reis et al. [31] Meta-analysis (14 RCT; n = 1.527)	“n” between groups not reported; Healthy participants (n = 7) Patients with IBS (n = 4) Patients with MDD (n = 1) Cancer patients (n = 1) Pregnant women (n = 1) Age ranged from 18 to 70 years	Probiotics: *L. casei Shirota YIT9029*; *L. rhamnosus JB-1*; *C. butyricum*; *L. helveticus* and *B. longum R0175*. Forms: capsules, fermented milk/yogurt, powder; Duration: 2–12 weeks.	Anxiety: SMD = −0.12 [95%CI: −0.29 to 0.05]; *p* = 0.151, indicating that probiotic administration did not result in reduction in anxiety; Subgroup: Anxiety in healthy: SMD = −0.10 [95%CI: −0.33 to 0.13]; *p* = 0.283; Clinical participants: SMD = −0.33 [95%CI: −1.08 to 0.43]; *p* = 0.312.
Ng et al. [17] Meta-analysis (10 RCT; n = 1349)	“n” between groups not reported; Healthy participants (n = 6) Stressed Workers (n = 1) Patients with MDD (n = 2) Patients with IBS (n = 1) Age ranged from 19.7 to 76.0 years	Multi-strain and mono-strain Probiotics: *L. acidophilus; L. casei*, *B. bifidum*; *L. gasseri SBT2055* and *B. longum SBT2928*; Forms: capsules, fermented milk/yogurt, Powder; Duration: 30 d–12 weeks.	Global mood effect: SMD = −0.12 [95%CI −0.261–0.004]; *p* = 0.059; Subgroup: **Significant effect among participants with MDD symptoms** **SMD = −0.684 [95%CI −1.296 to −0.071]; *p* = 0.029.** Healthy participants: SMD = −0.09 [95%CI −0.235 to 0.034]; *p* = 0.146; No adverse events were reported across the 10 studies.
Liu et al. [32] Meta-analysis (34 RCT n = 429, Prebiotics; n = 2731, Probiotics)	7 RCT Prebiotics Patients with IBS (n = 2) Patients with MDD (n = 1) Health Community (n = 3) 29 RCT Probiotics; Patients with IBS (n = 4) Patients with MDD (n = 3 Health Community (n = 15) Clinical patients (n = 5)	Prebiotics: GOS, FOS, FOS-enriched insulin; Dose: not reported; Duration: 4 hours to 8 weeks; Probiotics: *B. bifidum*, *L. acidophilus*, *L. casei*; *B. breve*, *B. longum*; *Streptococcus thermophilus*; *L. paracasei*; *L. reuteri*; *L. rhamnosus*; *L. bulgaricus*; Duration: 8 days-45 sem;	Prebiotics (General): MDD: SMD = −0.08 [95%CI −0.30 to 0.15]; *p* = 0.51; Anxiety: SMD = 0.12 [95%CI −0.03 to 0.27]; *p* = 0.11; Probiotics (General): **MDD: SMD = −0.24 [95%CI −0.36 to −0.12]; *p* < 0.01;** **Anxiety: SMD = −0.10 [95%CI −0.19 to −0.01]; *p* = 0.03.** Subgroup (Clinical Sample): **MDD: SMD = −0.73, [95% CI−1.02 to −0.44] *p* < 0.001; I^2^ = 48.2%.** **Anxiety: SMD = −0.11 [95%CI −0.20 to −0.01; *p* = 0.03; I^2^ = 5.0%.** **Duration > 4 weeks: SMD = −0.28 [95%CI −0.44 to −0.13]; *p* < 0.001.**
Goh et al. [33] Meta-analysis (19 RCT; n = 1901)	n = 1030 EXP; n = 871 PLA. Healthy participants (n = 9) Clinical patients (n = 2) Patients with MDD (n = 4) Patients with IBS (n = 2) Fibromyalgia patients (n = 1) Pregnant women (n = 1) Age ranged from 19.8 to 70.9 years;	Probiotics: *L. acidophilus*, *L. casei*, *L. helveticus*, *L. rhamnosus*, *L. reuteri*, *L. plantarum*, *B. longum*, *B. breve*, *B. bifidum*; Forms: capsules, sachets, tablets, fermented milk/yogurt, powder; Duration: 30 days–24 weeks; Single (n = 7) and multi-strain (n = 12).	**Global: SMD = −0.31 [95%CI −0.56 to −0.07]; *p* = 0.01; I^2^ = 82%.** Subgroups: **MDD: SMD = −0.75 [95%CI −1.09 to −0.41]; *p* < 0.001;** Clinical patients: NS; SMD = −0.26 [95%CI −0.70 to 0.17]; *p* = 0.24; Healthy population: NS; SMD = −0.25 [95%CI −0.60 to 0.11]; *p* = 0.17. **Single-strain vs. multi-strain: only multi-strain was significant** Safety: no differences in discontinuation rates (RR = 0.90; *p* = 0.62); Adverse events were comparable, except for increased abdominal discomfort in the probiotic group (*p* < 0.05).
Nikolova et al. [34] Meta-analysis (3 RCT; n = 229)	“n” between groups not reported; Patients with MDD (n = 3) Age ranged from 18 to 50 years	Probiotics: *L. acidophilus*; *L. casei*; *B. bifidum*; *L. helveticus B. longum*; Forms: capsules, sachets Duration: 8 weeks	Significant effect of probiotics on ↓ depressive symptoms; **SMD = −0.52 [95%CI** −**0.82 to −0.21]; *p* = 0.03; I^2^ = 88.3**
Zagórska et al. [35] Meta-analysis (23 RCT; n = 2726)	Patients with MDD (n = 16) Patients with Anxiety (n = 14) Patients with Schizophrenia (n = 4) Stressed patients (n = 5) Age ranged from 18 to 74 years	Probiotics: *Lactobacillus* and *Bifidobacterium* (single- and multi-strain); some studies combining ≥6 strains. Duration: 4–24 weeks.	**Global effect: SMD = −0.35 [95%CI −0.59 to −0.12]; *p* = 0.006; I^2^ = 79%.** **MDD: SMD = −0.87 [95%CI −1.66 to −0.09]; *p* = 0.03).** Healthy participants: NS; SMD = −0.16 [95%CI −0.34 to 0.02]; *p* = 0.09. Anxiety: NS; SMD = −0.16 [95%CI −0.43 to 0.11]; *p* = 0.24; I^2^ = 87%. Stress: NS; SMD = −0.05 [95%CI −0.34 to 0.24]; *p* = 0.75; I^2^ = 25%.
Chao et al. [36] Meta-analysis (10 RCT; n = 773)	“n” between groups not reported; Patients with MDD (n = 4) Stressed patients (n = 6) Age ranged from 18 to 65 years	Probiotics: *L. helveticus*; *B. longum*; *L. plantarum*; *L. acidophilus*; *L. casei*; *Bifidobacterium bifidum*; *L. rhamnosus*; *L. casei Shirota*; Duration: 6–24 weeks	MDD: SMD = −0.48 [95%CI −0.71 to −0.26]; *p* = 0.27; Anxiety: NS; SMD = 0.00 [95%CI −0.41 to 0.41]; *p* = 0.23; Healthy Patients with MDD: SMD= −3.52 [95%CI −5.68 to −1.35] *p* = 0.08; Healthy Patients with Stress: SMD = −0.73 [95%CI −4.31 to 2.86], *p* = 0.18.
Zhang et al. [37] Meta-analysis (7 RCT; n = 1198)	n = 463 EXP; n = 735 PLA Healthy participants (n = 4) Clinical patients (n = 1) Stressed patients (n = 2) Age range: Not reported	Probiotics: *L. helveticus* and *B. longum*; *L. helveticus*; *B. bifidum*; *L. reuteri*; *L. rhamnosus*; *L. bulgaricus*; *S. thermophilus*; *L. plantarum*; Forms: ProbioStick; capsule; tablet; yogurt and powder. Duration: 30 days–24 weeks	**Stress-related anxiety: SMD = −0.14 [95%CI −0.27 to −0.01]; *p* = 0.03; I^2^ = 0%** Subgroup: Single-strain: NS; SMD = −0.12 [95%CI −0.26 to 0.02]; *p* = 0.09; I^2^ = 0% Multi-strain: NS; SMD = −0.32 [95%CI −0.30 to 0.05]; *p* = 0.11; I^2^ = 0% Short-term: NS; SMD = −0.13 [95%CI −0.30 to 0.05]; *p* = 0.17; I^2^ = 0% Long-term: NS; SMD = −0.16 [95%CI −0.36 to 0.03]; *p* = 0.09; I^2^ = 0%;
Amirani et al. [38] Meta-analysis (12 RCT; n = 656)	n = 330 EXP; n = 326 PLA MDD patients (n = 6) Alzheimer patients (n = 3) Stressed patients (n = 2) Patients with Schizophrenia (n = 1) Age ranged from 36.2 to 78.5 years	Probiotics: *Lactobacillus* or *Bifidobacter* strains: *L. acidophilus*, *L. casei*, *B. bifidum*, *B. longum*, *L. fermentum*, *L. plantarum*, *B. lactis*, *L. bulgaricus*, *L. rhamnosus*, *B. breve*; Form: capsules, sachets, and probiotic-containing foods; Duration: 6–12 weeks	**MDD: WMD = −9.60 [95%CI −10.0 to −9.1]; *p* < 0.05;** NS for age (<40 or >40 years); CRP Levels: NS; WMD = −1.59 [95%CI −2.2 to −0.97]; *p* > 0.05; **TNF-α levels: WMD = −0.12 [95%CI −0.20 to −0.05]; *p* < 0.05;** IL-1B levels: NS; WMD = −0.34 [95%CI −1.43 to 0.74]; *p* > 0.05; **IL-10 levels: WMD = −0.29 [95%CI −0.48 to −0.11]; *p* < 0.05**
Hofmeister et al. [39] Meta-analysis (62 RCT; n = 5059) Probiotics (n = 44); Prebiotics (n = 5); Synbiotic (n = 6)	Probiotics: Patients with MDD (n = 9) Healthy participants (n = 9) Patients with IBS (n = 9) Clinical patients (n = 8) Bipolar patients (n = 2) Premenopausal female (n = 1) Pregnant female (n = 2) Multiple sclerosis patients (n = 3) Fibromyalgia patients (n = 1) Prebiotics: Patients with MDD (n = 3) Healthy participants (n = 2) Synbiotics: Healthy participants (n = 6)	Probiotics: *Lactobacillus* (n = 41); *Bifidobacterium* (n = 29); Other: *Bacillus*, *Clostridium*, *Lactococcus*, *Streptococcus*, *Weisella*, and *Lacticaseibacillus*. Prebiotics: compounds in food that induce growth or activity of gut microbiota Duration: 4–52 weeks.	Probiotics: **MDD: SMD = −0.78 [95%CI −0.19 to −1.37]; I^2^ = 89.9%; *p* < 0.05;** **Without depression: SMD = −0.31 [95%CI −0.15 to −0.46]; I^2^ = 74.4%; *p* < 0.05;** Prebiotics: **MDD: SMD = −0.39 [95%CI−0.04 to −0.73]; I^2^ = 26.6%; *p* < 0.05;** Without depression: NS; SMD = −0.13 [95%CI −0.23 to −0.48]; Synbiotics: Without depression: NS; SMD = −0.68 [95%CI −0.36 to 1.00]; I^2^ = 44.0%;
Nikolova et al. [40] Meta-analysis (7 RCT; n = 404)	“n” between groups not reported; Patients with MDD (n = 7) Age ranged from 35 to 43 years (52–85% female)	Probiotics: *Lactobacillus* and *Bifidobacterium* strains (mostly multi-strain formulations). Examples include *L. acidophilus*, *L. casei*, *B. bifidum*, *L. helveticus*, *B. longum*, and *L. plantarum*; Duration: 6–8 weeks.	**Global: SMD = −0.58 [95%CI −0.19 to −0.97; I^2^ = 73%, *p* < 0.01;** **Adjunctive (add-on): SMD = −0.83 [95%CI −0.49 to −1.17]; *p* < 0.01;** Stand-alone: SMD = −0.02 [95%CI −0.34 to −0.30], NS; excluding high risk of bias study: SMD = −0.67 [95%CI −0.40 to −0.95]; *p* > 0.05
Cohen Kadosh et al. [41] Meta-analysis (10 RCT; n = 1503) Probiotics (n = 6) Prebiotics (n = 4)	“n” between groups not reported; Healthy students (n = 9) Children participants (n = 1) Age ranged from 18 to 30 years	Probiotics: *B. longum*, *B. Infantis*, *L. acidophilus*, *L. plantarum*, *L. paracasei*, *L. bulgaricus*, *S. thermophilus*, *L. casei Shirota*, Fermented Ginseng, Saccharomyces boulardii; Prebiotics: FOS, GOS; administered once to twice a day Forms: capsule, sachet, tablet, yogurt; liquid; Duration: 14–84 days	Anxiety: NS; SMD = −0.03 [95%CI −0.21 to 0.14]; *p* = 0.32; I^2^ = 12%;
El Dib et al. [42] Meta-analysis (16 RCT and quasi-RCTs.; n = 1125)	n = 754 EXP; n = 806 PLA Patients with MDD (n = 7) Patients with Anxiety (n = 4) Age ranged from 18 to 65 years	Probiotics: *L. plantarum*, *L. helveticus*, *L. rhamnosus*, *L. casei*, *L. casei Shirota*, *L. paracasei*, *L. plantarum*, *bulgaricus*, *delbrueckii bulgaricus*, *B. acidophilus; B. longum*, *B. bifidum*, *B. breve*, *B. Infantis*; *Sacchar omyces boulardii*; *S. salivarius thermophilus*; and *S. thermophilus*; Doses: NR; Forms: capsule, sachet, tablet, bottle; Duration: 4–24 weeks	**MDD (BDI): SMD = −0.35 [95%CI −0.71 to −0.02]; *p* = 0.02; I^2^ = 21%;** MDD (DASS): SMD = 0.18 [95%CI −0.08 to 0.45]; *p* = 0.16; I^2^ = 0%; MDD (MADRS): SMD = −0.34 [95%CI −1.43 to 0.74]; *p* = 0.56; I^2^ = 87%; **Anxiety (STAI): SMD = −0.63 [95%CI −1.00 to −0.25]; *p* = 0.01; I^2^ = 24%;** Anxiety (BAI): SMD = −0.28 [95%CI −0.59 to 0.03]; *p* = 0.06; I^2^ = 0%; Anxiety (DASS-A): SMD = −0.18 [95%CI −0.52 to −0.16]; *p* = 0.83; I^2^ = 74%; Stress: (DASS-S): SMD = 0.07 [95%CI −0.27 to 0.42]; *p* = 0.64; I^2^ = 34%;
Misera et al. [43] Meta-analysis (10 RCT; n = 603)	All patients with MDD Age ranged from 26,33 to 50,2 years	Probiotics: *L. helveticus*; *B. longum*; *B. bifidum*; *B. lactis*; *L. acidophilus*; *L. casei*; *L. paracasei*; *L. plantarum*; *L. salivarius*; *L. lacti*; Duration 28–62 days	**MDD: SMD** **= −0.292 [95%CI −0.57 to −0.007]; *p* < 0.044;** Subgroup: **BDI: SMD** **= −0.482 [95%CI −0.85 to −0.10]; *p* < 0.011**
Zhu et al. [54] Meta-analysis (15 RCT; n = 1345)	n = 675 EXP, n = 670 PLA; Patients with MDD (n = 7) Healthy participants (n = 5) Patients with IBS (n = 2) Clinical patients (n = 1) Age ranged from 20 to 75 years	Probiotics: *L. acidophilus*, *L. casei*, *L. helveticus*, *L. rhamnosus*, *L. plantarum*, *L. pentosus*, *B. bifidum*, *B. longum*, *B. breve*, *B. infantis*, *B. lactis*, *Bacillus coagulans*; Forms: capsule, sachet, tablet, Yogurt; Duration: 90 days–20 weeks.	**Global: SMD = −0.19 [95%CI −0.01 to −0.37]; *p* = 0.044; I^2^ = 59.7%.** Subgroups: **<60 years: SMD = −0.36 [95%CI −0.14 to −0.58]; *p* = 0.002; I^2^ = 57.5%;** ≥60 years: NS; SMD = −0.13 [95%CI −0.31 to −0.04]; *p* > 0.05. **Clinical MDD (n = 10): SMD = −0.46 [95%CI *p* < 0.001; I^2^ = 47.3%.** Healthy (n = 9): NS; SMD = −0.10 [95%CI −0.23 to −0.02]. **<8 weeks (n = 9): SMD = −0.30 (95%CI −0.12 to −0.47; *p* < 0.001; I^2^ = 27%);** ≥8 weeks (n = 10): NS; SMD = 0.08; 95%CI −0.20–0.35; *p* = 0.59; I^2^ = 67.6%. **Multi-strain (n = 9): SMD = −0.17 [95%CI −0.01 to −0.32]; *p* = 0.031;** Single-strain (n = 10): NS; SMD = 0.18 *p* = 0.34; I^2^ = 78.8%. **Solid forms: SMD = −0.27 [95%CI 0.06–0.48]; *p* = 0.01;** Liquid forms (yogurt/milk): NS; SMD = −0.17 [95%CI −0.57 to −0.23].
Le Morvan de Sequeira et al. [16] Meta-analysis, (30 RCT; n = 2595)	“n” between groups not reported Patients with MDD (n = 8) Patients with Anxiety (n = 5) Stressed patients (n = 7) Schizophrenia (n = 3) Age ranged from 36.2 (22.8–51.4) 62.9% were women	Probiotics: *L. plantarum*, *L. rhamnosus*, *L. gasseri*; *L. helveticus*, *L. reuteri*, *L. paracasei Lpc-37*, *L. casei Shirota*, *L. pentosus strain b240*, *L. casei YIT 9029*, *L. Shirota YIT9029*, *L. helveticus strain CM4*, *L. paracasei YIT9029 B. breve*, *B. breve A1*, Single strains (n = 16), Multi-strains (n = 14); Forms: Capsules, Tablets/pills, liquids/yogurt, powder Duration: 4–24 weeks.	**Depression: SMD = −0.37 [95%CI −0.55 to −0.20]; *p* ≤ 0.0001; I^2^ = 48%;** Anxiety: SMD = −0.30 [95%CI −0.60 to 0.01; *p* = 0.06; I^2^ = 86%; **Distress: SMD = −0.33 [95%CI −0.53 to −0.13]; *p* = 0.001; I^2^ = 36%;** Anxiety PSS: SMD = −0.17 [95%CI −0.33 to 0.00]; *p* = 0.05; I^2^ = 0% **Mood (POMS): SMD = 0.17 [95%CI 0.10 to 0.24]; *p* < 0.0001; I^2^ = 0%;**
Zhang et al. [27] Meta-analysis (13 RCT; n = 786)	n = 427 EXP; n = 359 PLA. Adults with MDD (n = 13) Age ranged from: 34.5–53.0 years ≥50% women in all studies	Probiotics (n = 9): *L. casei*, *L. acidophilus*, *L. helveticus*, *L. rhamnosus*, *L. plantarum*, *B. longum*, *B. bifidum*, *B. breve.* Prebiotics (n = 3): GOS, inulin. Synbiotic: (n = 1): multi-strains + fluoxetine. Duration: 3–24 weeks	**Global MDD: SMD = −0.34 [95%CI −0.45 to −0.22; *p* < 0.001; I^2^ = 28.7%;** Subgroup Analysis: **Mild MDD: SMD = −0.38 [95%CI −0.63 to −0.14]; *p* = 0.002; I^2^ = 20.8%;** **Moderate MDD: SMD = −0.39 [95%CI −0.54 to −0.24]; *p* < 0.001; I^2^ = 21.8%;** **<70% women: SMD = −0.49 [95%CI −0.68 to −0.30], *p* < 0.001);** **≥70% women: SMD = −0.21; [95%CI −0.38 to −0.05]; *p* = 0.011);** **Probiotics MDD: SMD = −0.35 [95%CI −0.47 to −0.22]; *p* < 0.001; I^2^ = 38.2%;** Prebiotics MDD: NS; SMD = −0.25 [95%CI −0.64 to 0.15]; *p* = 0.22; I^2^ = 28.7%; **Multi-strains: SMD = −0.27 [95%CI −0.43 to −0.10]; *p* = 0.002; I^2^ = 53.6%;** **Mono-strain: SMD = −0.42 [95%CI −0.62 to −0.22]; *p* < 0.001; I^2^ = 0** **≤4 weeks: SMD = −0.37 [95% CI −0.55 to −0.19]; *p* < 0.001;** **4–8 weeks: SMD = −0.32 [95%CI −0.51 to −0.14]; *p* = 0.001**
Lin et al. [44] Meta-analysis (13 RCT; n = 776)	n = 397 EXP, n = 379 PLA; Patients with MDD Multiple sclerosis patients (n = 2) Clinical patients Fibromyalgia patients (n = 1) Patients with subclinical symptoms;	Probiotics: *Lactobacillus*, *Bifidobacterium*, *Streptococcus*, *Bacillus*, including multi-strain and single-strain formulas; Forms: capsules, powder, yogurt, tablets; Duration: 4–20 week.	**BDI-II global: MD = −1.98 (95%CI −3.14 to −0.82; *p* < 0.001; I^2^ = 76%).** Subgroups: MDD: NS; MD = −1.66 [95%CI − 3.33 to 0.02]; *p* = 0.05; I^2^ = 0%; **≥40 years: MD = −2.80 [95%CI −4.17 to −1.43]; *p* < 0.001;** <40 years: NS; MD = −0.40 [95%CI −1.52 to 0.71]; *p* = 0.48; **≤8 weeks: MD = −3.28 [95%CI −5.55 to −1.00]; *p* = 0.005;** **>8 weeks: MD = −1.20 [95%CI −2.35 to −0.05]; *p* = 0.04;**
Zhao et al. [45] Meta-analysis (23 RCT; n = 2035)	n = 1030 EXP, n = 1005 PLA Patients with MDD(n = 3) Patients with Anxiety (n = 29) Healthy adults(n = 8) Other comorbidities (n = 10)	Probiotics: *L. acidophilus*; *B. longum*; *L. helveticus*; *B. bifidum*; *L. rhamnosus*; Prebiotics: inulin; FOS; GOS; Synbiotics: *L. acidophilus and* inulin; *B. bifidum* and FOS Forms: Capsules; Sachets; Spray-Dried Powder; Pills; Fermented Liquids; Freeze-Dried Powder Duration: 4–24 week.	**Global Anxiety: SMD = −0.16 [95%CI −0.26 to −0.05]; *p* < 0.01;** Subgroup: **STAI-S: SMD = −0.29 [95%CI −0.56 to −0.02]; *p* = 0.04, I^2^ = 36%;** **BAI: SMD = −0.26 [95%CI −0.49 to −0.03]; *p* = 0.03, I^2^ = 0%;** **Mental health: SMD = −0.29 [95%CI −0.54 to −0.04]; *p* = 0.02;** **Anxiety (IBS): SMD = −0.14 [95%CI −0.27 to −0.01]; *p* = 0.03;** Anxiety without IBS: SMD = −0.16 [95%CI −0.32 to 0.00]; *p* = 0.05; **Low dose: SMD = −0.16 [95%CI −0.29 to −0.03]; *p* = 0.04; I^2^ = 18%;** **Multi-strain: SMD = −0.19 [95%CI −0.32 to −0.07]; *p* < 0.01; I^2^ = 50%;** **≥8 weeks: SMD = −0.14 [95%CI −0.27 to −0.01] *p* = 0.04 I^2^ = 39%** **<8 weeks: SMD = −0.20 [95%CI −0.39 to −0.01]; *p* = 0.05; I^2^ = 11%** **Compared with probiotics, synbiotics had a greater positive effect on Synbiotic (Anxiety): SMD = −0.71 [95%CI −1.04 to −0.38]; *p* < 0.01; I^2^ = 0%** **Prebiotic (Anxiety): SMD = 0.08 [95%CI −0.29 to 0.45]; *p* = 0.66; I^2^ = 16%**
Huang et al. [46] Meta-analysis (13 RCT; n = 768)	“n” between groups not reported Patients with MDD (n = 3) Patients with Anxiety (n = 1) Patients with general depression (n = 1) Healthy adults (n = 4) Condition not detailed (n = 1)	Probiotics: *Bifidobacterium* (*various*) Forms: capsules; Dry powder; Freeze-dried; Bibiotic tetrad tablets; spray-dried, Duration: 60 days–12 weeks	**General: MD** **= −0.49 [95%CI −0.71 to −0.26]; *p* < 0.0001;** Subgroup analysis **Antidepressants (n = 4): MD** **= −0.82 [95%CI −1.07 to −0.58]; *p* < 0.00001;** **Bifidobacterium (n = 9): MD** **= −0.33 [95%CI −0.59 to −0.08]; *p* = 0.008;** Subgroup by type of depression **MDD (n = 3): MD** **= −0.66 [95%CI −1.01 to −0.32]; *p* = 0.0002;** **MDD (n = 6) + healthy volunteers (n = 4) with subclinical depressive symptoms: MD** **= −0.44 [95%CI −0.72 to −0.16]; *p* = 0.002;**
Rahmannia et al. [47] Meta-analysis (RCT 12; n = 707)	n = 355 EXP, n = 352 PLA; Patients with MDD (n = 12) Patients with obesity (n = 1)	Probiotics: *L. acidophilus*; *L. paracasei*; *L. casei*; *L. plantarum*; *L. salivarius*; *B. bifidum*; *B. lactis*; *B. breve*; *B. longum*, with additional nutrients like magnesium, methionine, vitamin B7. Duration: 4–12 weeks	**BDI: MD = −2.69 [95%CI −4.22 to −1.16]; *p* = 0.00; I^2^ = 0.0%;** HAMD: MD = −1.40, [95%CI −3.29 to 0.48]; *p* = 0.14; I^2^ = 69%; DASS: MD = −2.57 [95%CI −0.71 to 5.80]; *p* = 0.12; I^2^ = 0.0%; MADRS: MD = −2.41 [95%CI −9.18 to 5.73]; *p* = 0.56; I^2^ = 86%;
Asad et al. [48] Meta-analysis (20 RCT; n = 1401)	“n” between groups not reported Patients with MDD Patients with Anxiety Patients with IBS Patients with Bipolar Disorder Age ranged from 21 to 53 years. 54% women.	Probiotics: *L. helveticus*, *L. acidophilus*, *L. casei*, *L. rhamnosus*, *L. plantarum*, *B. longum*, *B. bifidum*, *B. breve*, *B. coagulans*, *C. butyricum*, multi-strains, and mono-strains. Duration: 3–24 weeks	**Global MDD: SMD** **= −0.96 [95%CI −1.31 to −0.61] *p* < 0.05; I^2^ = 85%;** **Global Anxiety: SMD** **= −0.59 [95%CI −0.98 to −0.19]; I^2^ = 79%;** Subgroup Depression: **Stand-alone MDD: SMD** **= −0.95 [95%CI −1.50 to −0.60]; *p* < 0.05;** **Diagnosed MDD: SMD** **= −1.09 [95%CI −1.54 to −0.64]; *p* < 0.05; I^2^ = 85%;** **Multi-strain MDD: SMD** **= −0.92 [95%CI −1.46 to −0.38]; *p* < 0.05; I^2^ = 90%** **Single-strain MDD: SMD** **= −1.03 [95%CI−1.41 to −0.65]; *p* < 0.05; I^2^ = 66%** **<8 weeks MDD: SMD** **= −1.54 [95%CI −2.18 to −0.90]; *p* < 0.05; I^2^ = 85%;** **>8 weeks MDD: SMD** **= −0.59 [95%CI −0.86 to −0.33]; *p* < 0.05; I^2^ = 63%;** Subgroup Anxiety: **Diagnosed Anxiety: SMD** **= −1.05 [95%CI −1.77 to −0.33]; I^2^ = 76.3%;** **Multi-strain Anxiety: SMD** **= −0.50 [95%CI −0.95 to −0.05]; *p* < 0.05; I^2^ = 90%;** **Single-strain Anxiety: SMD** **= −0.91 [95%CI −1.81 to −0.00]; *p* < 0.05; I^2^ = 66%;** **<8 weeks Anxiety: SMD** **= −0.88 [95%CI−1.68 to −0.09]; *p* < 0.05; I^2^ = 89%** **>8 weeks Anxiety: SMD** **= −0.31 [95%CI −0.53 to −0.08]; I^2^ = 0%;**
Sulaiman et al. [49] Meta-analysis (12 RCT; n = 553)	n = 266 EXP; n = 286 PLA; Unspecified population; however, with patients with MDD. Age ranged from 18 to 79 years;	Probiotics: *L. acidophilus*, *L. casei*, and *B. bifidum*; *B. bifidum*, *B. lactis*, *L. acidophilus*, *L. brevis*; *L. paracasei*; *S. thermophilus*, *B. breve*, *B. longum*, *B. Infantis*, *L. plantarum*, *B. subtilis.*	**Global MDD: SMD = −0.55 [95%CI −1.06 to −0.05]; *p* < 0.05** **HAM-D: SMD = −0.92 [95%CI −1.84 to −0.11]; *p* = 0.04**
Zhao et al. [50] Network meta-analysis (42 RCT; n = 13,050)	22 different types of MDD interventions were included in this review	Probiotics: Non-reported Duration: 6–24 weeks	Global SMDs = −0.16 [95%CI −0.30 to −0.04]; *p* > 0.05; Subgroups (probiotics superior): **Brexpiprazole: SMD = −0.42 [95%CI −0.68 to −0.17];** **Cariprazine: SMD = −0.44 [95%CI −0.69 to −0.24];** **Citalopram: SMD = −0.37 [95%CI −0.66 to −0.07];** **Duloxetine: SMD = −0.26 [95%CI −0.51 to −0.04];** **Desvenlafaxine: SMD = −0.38 [95%CI −0.63 to −0.14];** **Ketamine: SMD = −0.32 [95%CI −0.66 to −0.01];** **Venlafaxine: SMD = −0.47 [95%CI −0.73 to −0.23];** **Vilazodone: SMD = −0.37 [95%CI −0.61 to −0.12];** **Vortioxetine: SMD = −0.39 [95%CI −0.63 to −0.15];** **Placebo: SMD = −0.62 [95%CI −0.86 to −0.42];**
Zandifar et al. [51] Meta-analysis (53 RCT; Anxiety: n = 4295; MDD: n = 3179; Cognitive: n = 915)	Anxiety (n = 2194 EXP, n = 2101 PLA, Depression (n = 1603 EXP, n = 1576 PLA) Cognitive Function (n = 470 EXP, n = 445 PLA)	Probiotic, Prebiotic, or Synbiotic, The types and strains were not specified. Probiotics and vitamin D.	**Global Anxiety: SMD** **= −0.29 [95%CI −0.57 to −0.02]; *p* < 0.05, I^2^ = 92%;** **Global MDD: SMD** **= −0.29 [95%CI −0.57 to −0.02]; *p* < 0.05; I^2^ = 91%** **Cognitive function: SMD** **= 0.48 [95%CI 0.17 to 0.80]; *p* < 0.01; I^2^ = 77%;**
Cheng et al. [52] Meta-analysis (12 RCT; n = 1052)	Patients with MDD (n = 1) Clinical patients (n = 7) Multiple sclerosis patients (n = 1) Patients with IBS (n = 1) Children (n = 1) Patients with ADHD (n = 1)	Mixed Probiotics: *B. bifidum F-35*, *B. longum CCFM729*, *L. plantarum CCFM639*, *L. acidophilus CCFM137*, *L. casei CN1566*, *L. reuteri DSM17938*, *L. rhamnosus CCFM10281* or *L. reuteri.*	**Global MDD: SMD = −0.44 [95%CI −0.72 to −0.16]; *p* < 0.001; I^2^ = 78%** Subgroup: **Multiple strains: SMD = −0.56 [95%CI −0.97 to −0.15]; *p* <0.00001; I^2^ = 82%;** *L. reuteri* vs. Multi-strain: SMD = −0.20 [95%CI −0.45 to 0.06]; *p* = 0.13; I^2^ = 51% ***L. reuteri* vs. PLA: SMD = −0.39 [95%CI −0.74 to −0.03]; *p* < 0.05; I^2^ = 83%** **<60 years: SMD = −0.52 [95%CI −0.88 to −0.15; *p* = 0.0001; I^2^ = 81%;** >60 years: SMD = −0.22 [95%Cl −0.57 to 0.13]; *p* = 0.12; I^2^ =52%; **Female ≥50%: SMD = −0.45 [95%CI −0.78 to −0.13; *p* = 0.00001; I^2^ = 82%;** Female < 50%: SMD = −0.41 [95%CI −0.82 to 0.00]; *p* = 0.67; I^2^ = 0%;
Moshfeghinia et al. [53] Meta-analysis (19 RCT) Depression: (n = 1405) Anxiety (n = 481)	Depression (n = 882 EXP, n = 523 PLA) Anxiety (n = 237 EXP, n = 244 PLA) Patients with MDD (n = 9) Non-specific depressed (n = 5) Clinical patients (n = 4) Pregnant female (n = 1)	Probiotic: *L. plantarum*; *L. rhamnosus*; *L. casei*; *L. acidophilus*; *L. gasseri*; *L. reuteri*; *B. bifidum*; *B. longum*; *B. lactis*; *S. thermophilus*; *L. mesenteroides*; Prebiotics: Inulin; FOS; GOS; Synbiotic: *L. plantarum* and inulin; *B. bifidum* and FOS; *L. rhamnosus* and GOS Drinks, capsules, sachet containing freeze-dried; Duration: 1–24 weeks	**Global MDD: SMD = −1.76 [95%CI −2.42 to −1.10]; *p* < 0.05; I^2^ = 96.29%;** Global Anxiety: SMD = −1.60 [95%CI −2.83 to 0.36]; *p* > 0.05; I^2^ = 96.9%; Analysis of the subgroups for depression: **BDI: SMD= −1.22 [95%CI −1.87 to −0.58]; *p* < 0.001; I^2^ = 88.5%;** **MADRS: SMD = −3.25 [95%CI −6.60 to 0.10]; *p* < 0.001; I^2^ = 97.0%;** **HDRS: SMD = −2.09 [−3.54 to −0.64]; *p* < 0.001; I^2^ = 97.7%;** **Multi-strain: SMD = −1.56 [95%CI −2.28 to −0.85]; *p* < 0.001; I^2^ = 96.0%;** **≤40 years: SMD = −1.18 [95%CI −1.83 to −0.53]; *p* < 0.001; I^2^ = 93.2%;** **>40 years: SMD = −2.54 [95%CI −3.80 to −1.27]; *p* < 0.001; I^2^ = 97.0%;** **Probiotics: SMD = −1.96 [95%CI −2.75 to −0.64]; *p* < 0.001; I^2^ = 96.9%;** **Prebiotic: SMD = −0.78 [95%CI −1.63 to −0.07]; *p* < 0.001; I^2^ = 80.3%** **Synbiotics: SMD = −1.25 [95%CI −1.91 to −0.58]; *p* < 0.001;** Analysis of the subgroups for anxiety STAI: SMD = −0.14 [95%CI −0.43 to 0.16]; *p* = 0.85; I^2^ = 0%; **BAI: SMD = −2.45 [95%CI −3.18 to −1.72]; *p* < 0.001; I^2^ = 0%;** **Multi-Strain: SMD = −0.81 [95%CI −1.62 to 0.00]; *p* < 0.001; I^2^ = 92.0%;** **≤40 years: SMD = −1.36 [95%CI −2.66 to −0.07]; *p* < 0.001; I^2^ = 96.9%;** **>40 years: SMD = −3.48 [95%CI −4.28 to −2.68]; *p* < 0.001;**

Clinical Trials and Statistics: ECR/RCT—Randomized Controlled Trial; EXP—Experimental Group (intervention); PLA—Placebo; CON—Control; MD—Mean Difference; SMD—Standardized Mean Difference; CI/95% CI—95% Confidence Interval; NS—Not Significant; RR—Relative Risk; Hedges’ g/Cohen’s d—Effect size measures; and I^2^—Heterogeneity statistic in meta-analyses. Disorders and Clinical Conditions: MDD—Major Depressive Disorder; TRD—Treatment-Resistant Depression; IBS—irritable bowel syndrome; CFS—chronic fatigue syndrome; MS—Multiple Sclerosis; CAD/CHD—coronary artery disease; BD—bipolar disorder; GAD—Generalized Anxiety Disorder; PTSD—Post-Traumatic Stress Disorder; AUD—Alcohol Use Disorder; ASD—autism spectrum disorder; ADHD—Attention-Deficit/Hyperactivity Disorder; MCI—Mild Cognitive Impairment; Psychometric Scales: BDI/BDI-II—Beck Depression Inventory; HAM-D/HAMD-17/HAMD-24—Hamilton Depression Rating Scale; MADRS—Montgomery–Åsberg Depression Rating Scale; QIDS-SR16—Quick Inventory of Depressive Symptomatology—Self-Report, 16 items; CES-D—Center for Epidemiologic Studies Depression Scale; GDS/GDS-SF/GDS-15—Geriatric Depression Scale; DASS-21/DASS-42/DASS-D/DASS-A/DASS-S—Depression, Anxiety and Stress Scale; HADS/HADS-D/HADS-A—Hospital Anxiety and Depression Scale; PHQ-9—Patient Health Questionnaire-9; EPDS—Edinburgh Postnatal Depression Scale; BAI—Beck Anxiety Inventory; HAM-A—Hamilton Anxiety Rating Scale; STAI—State-Trait Anxiety Inventory; PSS—Perceived Stress Scale; POMS—Profile of Mood States; HSCL-90/HSCL-90-R—Hopkins Symptom Checklist; LEIDS-r—Leiden Index of Depression Sensitivity—revised; GHQ/GHQ-28—General Health Questionnaire; MINI—Mini International Neuropsychiatric Interview; PANSS—Positive and Negative Syndrome Scale; BPRS—Brief Psychiatric Rating Scale; YMRS—Young Mania Rating Scale; RBANS—Repeatable Battery for the Assessment of Neuropsychological Status; MMSE—Mini-Mental State Examination; IBS-SSS—Irritable Bowel Syndrome Symptom Severity Scale; VSI—Visceral Sensitivity Index; Biomarkers: BDNF—Brain-Derived Neurotrophic Factor; CRP/hs-CRP—C-Reactive Protein (high-sensitivity); IL-6, IL-10—Interleukin 6 and 10; TNF-α—Tumor Necrosis Factor-alpha; IFN-γ—Interferon Gamma; 5-HT—Serotonin (5-Hydroxytryptamine); TPH2—Tryptophan Hydroxylase 2; NO—Nitric Oxide; GSH—Glutathione; TAC—Total Antioxidant Capacity. “B”—Bifidobacterium; “L”—Lactobacillus; “S”—Streptococcus; “C”—Clostridium; GOS—Galactooligosaccharide; and FOS—Fructooligosaccharides. Outcomes in bold represent positive results (*p* < 0.05).

## Data Availability

The original contributions presented in this study are included in the article/Supplementary Material. Further inquiries can be directed to the corresponding authors.

## Supplementary Materials

The following supporting information can be downloaded at https://www.mdpi.com/article/10.3390/ph19010156/s1: Supplementary Materials 1: Table S1. Electronic complete search strategy across the different databases; Table S2. List of included studies and their respective databases; Table S3. List of excluded articles and their reasons; Table S4. Methodological quality assessment of the included reviews using the AMSTAR 2 tool. Table S5. Justification for including SMD Global or the SMD subgroup for analysis. References [87,88,89,90,91] are cited in Supplementary Materials 1. Supplementary Materials 2: PRISMA 2020 Checklist.

## Abbreviations

The following abbreviations are used in this manuscript:

AMSTAR 2	A measurement tool to assess systematic reviews (version 2)
ACTH	Adrenocorticotropic hormone
BAI	Beck Anxiety Inventory
BDI	Beck Depression Inventory
BDNF	Brain-derived neurotrophic factor
CFU	Colony-forming unit
CI	Confidence interval
CRH	Corticotropin-releasing hormone
DSM-5	Diagnostic and Statistical Manual of Mental Disorders, Fifth Edition
GABA	Gamma-aminobutyric acid
GAD	Generalized anxiety disorder
GI Tract	Gastrointestinal tract
GOS	Galactooligosaccharide
HADS	Hospital Anxiety and Depression Scale
HAM-A	Hamilton Anxiety Rating Scale
HAM-D	Hamilton Depression Rating Scale
HPA Axis	Hypothalamic
Pituitary	Adrenal axis
HDAC	Histone deacetylase
IBS	Irritable bowel syndrome
IL-6	Interleukin-6
IL-10	Interleukin-10
KYN Pathway	Kynurenine pathway
MeSH	Medical subject headings
NE	Norepinephrine
PRISMA	Preferred Reporting Items for Systematic Reviews and Meta-Analyses
PROSPERO	International Prospective Register of Systematic Reviews
QOL	Quality of life
RCT	Randomized controlled trial
SCFAs	Short-chain fatty acids
SCID	Structured Clinical Interview for DSM Disorders
SMD	Standardized mean difference
SNRI	Serotonin–norepinephrine reuptake inhibitor
SSRI	Selective serotonin reuptake inhibitor
STAI	State-Trait Anxiety Inventory
TNF-α	Tumor necrosis factor–alpha
WHOQOL-BREF	World Health Organization Quality of Life–Brief Version
