# Supplementary material for: “Attacking” the Gut–Brain Axis with Psychobiotics: An Umbrella Review of Depressive and Anxiety Symptoms
Source: Pharmaceuticals (Basel). 2026 Jan 15;19(1):156. doi: 10.3390/ph19010156 (PMC12845323; doi:10.3390/ph19010156)
Supplement: Supplementary file 1 [file pharmaceuticals-19-00156-s001.zip › pharmaceuticals-4039435-supplementary 1.pdf]

Supplementary material 1

Table S1. Electronic complete search strategy among the different databases

**PubMed**
(psychobiotics OR probiotics OR prebiotics OR symbiotics) AND (depressive OR depressive disorder OR anxiety) AND Meta-Analysis
https://pubmed.ncbi.nlm.nih.gov/?term=%28psychobiotics+OR+probiotics+OR+prebiotics+OR+symbiotics%29+AND+%28depressive+OR+depressive+disorder+OR+anxiety%29+AND+Meta-Analysis&filter=pubt.meta-analysis&filter=pubt.systematicreview
**Web of Science (Clarivate)**
(psychobiotics OR probiotics OR prebiotics OR symbiotics) AND (depressive OR depressive disorder OR anxiety) AND systematic review
https://www.webofscience.com/wos/woscc/summary/4333b2eb-fa58-44b7-b5c6-61379fdf2d6f-017a7fb279/d300f176-c2d2-430d-9c92-0f58d56c510e-017a7fb280/relevance/1
**Scopus**
TITLE-ABS-KEY (psychobiotics OR probiotics OR prebiotics OR symbiotics) AND TITLE-ABS-KEY-AUTH (depressive OR depressive disorder OR anxiety) AND (TITLE-ABS-KEY (meta-analysis))
https://www.scopus.com/results/results.uri?sort=plf-f&src=s&sid=c4050769af8ef28e56ad7944b49bdd1c&sot=a&sdt=a&sl=175&s=TITLE-ABS-KEY%28psychobiotics+OR+probiotics+OR+prebiotics+OR+symbiotics%29+AND+TITLE-ABS-KEY-AUTH%28depressive+OR+depressive+disorder+OR+anxiety%29+AND+%28+TITLE-ABS-KEY%28meta-analysis%29%29&origin=searchadvanced&editSaveSearch=&txGid=734e6b124983c434ef9d6b58382d0216&sessionSearchId=c4050769af8ef28e56ad7944b49bdd1c&limit=200
**Scielo**
(psychobiotics) OR (probiotics) OR (prebiotics) OR (symbiotics) AND (depressive) OR (depressive disorder) OR (anxiety)
https://search.scielo.org/?q=%28psychobiotics%29+OR+%28probiotics%29+OR+%28prebiotics%29+OR+%28symbiotics%29+AND+%28depressive%29+OR+%28depressive+disorder%29+OR+%28anxiety%29&lang=pt&count=15&from=1&output=site&sort=&format=summary&fb=&page=1&filter%5Bsubject_area%5D%5B%5D=Health+Sciences&filter%5Bsubject_area%5D%5B%5D=Biological+Sciences&q=%28psychobiotics%29+OR+%28probiotics%29+OR+%28prebiotics%29+OR+%28symbiotics%29+AND+%28depressive%29+OR+%28depressive+disorder%29+OR+%28anxiety%29&lang=pt&page=1
**EBSCO**
TI (psychobiotics) OR TI (prebiotics or probiotics or synbiotics) AND TX (depression or anxiety) OR TX (depression or depressive disorder or depressive symptoms or major depressive disorder) AND TI (meta-analysis or systematic review)
https://research.ebsco.com/c/ambmat/search/results?q=TI%20(psychobiotics)%20OR%20TI%20(prebiotics%20or%20probiotics%20or%20synbiotics)%20AND%20TX%20(depression%20or%20anxiety)%20OR%20TX%20(depression%20or%20depressive%20disorder%20or%20depressive%20symptoms%20or%20major%20depressive%20disorder)%20AND%20TI%20(meta-analysis%20or%20systematic%20review)&autocorrect=y&db=fsr%2Cffh&expanders=concept&facetFilter=sourceTypes%3ANjA1TFI%3D&isDashboardExpanded=true&limiters=FT%3AY&qm=W3sidHlwZSI6ImZpZWxkIiwidmFsdWUiOiJwc3ljaG9iaW90aWNzIiwiY29kZSI6IlRJIn0seyJ0eXBlIjoibG9naWMiLCJ2YWx1ZSI6Ik9SIn0seyJ0eXBlIjoiZmllbGQiLCJ2YWx1ZSI6InByZWJpb3RpY3Mgb3IgcHJvYmlvdGljcyBvciBzeW5iaW90aWNzIiwiY29kZSI6IlRJIn0seyJ0eXBlIjoibG9naWMiLCJ2YWx1ZSI6IkFORCJ9LHsidHlwZSI6ImZpZWxkIiwidmFsdWUiOiJkZXByZXNzaW9uIG9yIGFueGlldHkiLCJjb2RlIjoiVFgifSx7InR5cGUiOiJsb2dpYyIsInZhbHVlIjoiT1IifSx7InR5cGUiOiJmaWVsZCIsInZhbHVlIjoiZGVwcmVzc2lvbiBvciBkZXByZXNzaXZlIGRpc29yZGVyIG9yIGRlcHJlc3NpdmUgc3ltcHRvbXMgb3IgbWFqb3IgZGVwcmVzc2l2ZSBkaXNvcmRlciIsImNvZGUiOiJUWCJ9LHsidHlwZSI6ImxvZ2ljIiwidmFsdWUiOiJBTkQifSx7InR5cGUiOiJmaWVsZCIsInZhbHVlIjoibWV0YS1hbmFseXNpcyBvciBzeXN0ZW1hdGljIHJldmlldyIsImNvZGUiOiJUSSJ9XQ%3D%3D&resetPageNumber=true&searchMode=boolean&searchSegment=all-results
**Cochrane Data Base**
(psychobiotics OR probiotics OR prebiotics OR symbiotics) AND (depressive OR depressive disorder OR anxiety) AND Meta-Analysis

Table S2. List of included studies and their respective databases.

Name	Country	Title	DOI	Source
Huang et al. [1]	China	Effect of Probiotics on Depression: A Systematic Review and Meta-Analysis of Randomized Controlled Trials	10.3390/nu8080483	WOS + PubMed + Scopus
Mckean et al. [2]	Australia	Probiotics and Subclinical Psychological Symptoms in Healthy Participants: A Systematic Review and Meta-Analysis	10.1089/acm.2016.0023	WOS + PubMed
Liu et al. [3]	China	Efficacy of probiotics on anxiety: A meta-analysis of randomized controlled trials	10.1002/da.22811	WOS + PubMed
Reis et al. [4]	United States of America	The anxiolytic effect of probiotics: A systematic review and meta-analysis of the clinical and preclinical literature	10.1371/journal.pone.0199041	Other sources
Ng et al. [5]	Singapore	A meta-analysis of the use of probiotics to alleviate depressive symptoms	10.1016/j.jad.2017.11.063	WOS + PubMed + Scopus
Liu et al. [6]	United States of America	Prebiotics and probiotics for depression and anxiety: A systematic review and meta-analysis of controlled clinical trials	10.1016/j.neubiorev.2019.03.023	PubMed
Goh et al. [7]	Taiwan	Effect of probiotics on depressive symptoms: A meta-analysis of human studies	10.1016/j.psychres.2019.112568	WOS + PubMed + Scopus
Nikolova et al. [8]	United Kingdom	Gut feeling: randomized controlled trials of probiotics for the treatment of clinical depression: Systematic review and meta-analysis	10.1177/2045125319859963	WOS + PubMed + Scopus
Zagórska et al. [9]	Poland	From probiotics to psychobiotics – the gut-brain axis in psychiatric disorders	10.3920/BM2020.0063	Other sources
Chao et al. [10]	China	Effects of Probiotics on Depressive or Anxiety Variables in Healthy Participants Under Stress Conditions or With a Depressive or Anxiety Diagnosis: A Meta-Analysis of Randomized Controlled Trials	10.3389/fneur.2020.00421	WOS + PubMed + Scopus
Zhang et al. [11]	China	Efficacy of probiotics on stress in healthy volunteers: A systematic review and meta-analysis based on randomized controlled trials	10.1002/brb3.1699	WOS + PubMed
Amirani et al. [12]	Iran	The effects of probiotic supplementation on mental health, biomarkers of inflammation and oxidative stress in patients with psychiatric disorders: A systematic review and meta-analysis of randomized controlled trials	10.1016/j.ctim.2020.102361	WOS + PubMed
Hofmeister et al. [13]	Canada	The effect of interventions targeting gut microbiota on depressive symptoms: a systematic review and meta-analysis	10.9778/cmajo.20200283	Other sources
Nikolova et al. [14]	United Kingdom	Updated Review and Meta-Analysis of Probiotics for the Treatment of Clinical Depression: Adjunctive vs. Stand-Alone Treatment	10.3390/jcm10040647	WOS + Scopus
Cohen Kadosh et al. [15]	United Kingdom	Psychobiotic interventions for anxiety in young people: a systematic review and meta-analysis, with youth consultation	10.1038/s41398-021-01422-7	WOS + PubMed

El Dib et al. [16]	Brasil	Probiotics for the treatment of depression and anxiety: A systematic review and meta-analysis of randomized controlled trials	10.1016/j.clnesp.2021.07.027	WOS + PubMed + Scopus
Misera et al. [17]	Poland	Effect of Psychobiotics on Psychometric Tests and Inflammatory Markers in Major Depressive Disorder: Meta-Analysis of Randomized Controlled Trials with Meta-Regression	10.3390/ph14100952	WOS + Scopus + Cochrane
Zhu et al. [18]	China	A psychobiotic approach to the treatment of depression: A systematic review and meta-analysis	10.1016/j.jff.2022.104999	Other sources
Le Morvan et al. [19]	Germany	Effect of Probiotics on Psychiatric Symptoms and Central Nervous System Functions in Human Health and Disease: A Systematic Review and Meta-Analysis	10.3390/nu14030621	WOS + PubMed
Zhang et al. [20]	China	Effect of prebiotics, probiotics, synbiotics on depression: results from a meta-analysis	10.1186/s12888-023-04963-x	WOS + PubMed
Lin et al. [21]	China	The effect and safety of probiotics on depression: a systematic review and meta-analysis of randomized controlled trials	10.1007/s00394-023-03184-y	WOS + PubMed + Scopus
Zhao et al. [22]	China	Effectiveness of probiotic/prebiotic/synbiotic treatments on anxiety: A systematic review and meta-analysis of randomized controlled trials	10.1016/j.jad.2023.09.018	WOS + PubMed
Huang et al. [23]	China	Efficacy of bifidobacterium-related preparations on depression: the first meta-analysis	10.3389/fpsyt.2024.1463848	WOS
Rahmannia et al. [24]	Iran	Strain-specific effects of probiotics on depression and anxiety: a meta-analysis	10.1186/s13099-024-00634-8	WOS + Scopus
Asad et al. [25]	United Kingdom	Effects of Prebiotics and Probiotics on Symptoms of Depression and Anxiety in Clinically Diagnosed Samples: Systematic Review and Meta-analysis of Randomized Controlled Trials	10.1093/nutrit/nuae177	WOS + PubMed
Sulaiman et al. [26]	Malaysia	An updated systematic review and appraisal of the pathophysiologic mechanisms of probiotics in alleviating depression	10.1080/1028415X.2025.2531357	WOS + PubMed
Zhao et al. [27]	China	Probiotics for adults with major depressive disorder compared with antidepressants: a systematic review and network meta-analysis	10.1093/nutrit/nuad171	WOS + PubMed + Scopus
Zandifar et al. [28]	Iran	The Effect of Prebiotics and Probiotics on Levels of Depression, Anxiety, and Cognitive Function: A Meta-Analysis of Randomized Clinical Trials	10.1002/brb3.70401	WOS + PubMed
Cheng et al. [29]	China	The efficacy and acceptability of Lactobacillus reuteri for the treatment of depression: A systematic review and meta-analysis	10.1016/j.genhosppsych.2025.05.004	WOS + PubMed + Scopus
Moshfeghinia et al. [30]	Iran	The impact of probiotics, prebiotics, and synbiotics on depression and anxiety symptoms of patients with depression: A systematic review and meta-analysis	10.1016/j.jpsychires.2025.05.053	WOS + PubMed + Scopus

Table S3. List of excluded articles and their reasons

Name	Title	DOI	Source	Reason
Budde et al. [31]	A 10 years update of effects of exercise on depression disorders—in otherwise healthy adults: A systematic review of meta-analyses and neurobiological mechanisms.	10.1371/journal.pone.0317610	EBSCO	Out of Scope
Moran et al. [32]	Efficacy of Probiotics, Prebiotics, and Symbiotics for the Treatment of Depression: A meta-review	10.17711/SM.0185-3325.2025.005	WOS + Scopus	Meta-Review Umbrella
Musazadeha et al. [33]	Probiotics as an effective therapeutic approach in alleviating depression symptoms: an umbrella meta-analysis	10.1080/10408398.2022.2051164	WOS + PubMed + Scopus	Meta-Review Umbrella
Goel et al. [34]	Association of Gut-Microbiome and mental health and effects of probiotics on psychiatric disorders: A Meta-analysis and systematic review	10.71480/nmj.v66i1.601	PubMed	Observational study
Breuling et al. [35]	Butyrate- and Beta-Hydroxybutyrate-Mediated Effects of Interventions with Pro- and Prebiotics, Fasting, and Caloric Restrictions on Depression: A Systematic Review and Meta-Analysis	10.3390/life14070787	WOS + PubMed	Metabolites as the main effect
Rokkas et al. [36]	Comparative effectiveness and safety of probiotics with psychotropic potential in mental health benefits in irritable bowel syndrome: a systematic review and network meta-analysis	10.1097/MEG.0000000000003062	PubMed	Irritable bowel syndrome as the main outcome
Haller et al. [37]	Complementary therapies for clinical depression: An overview of systematic reviews	10.1136/bmjopen-2018-028527	Scopus	Comparison of Multiple Interventions
He et al. [38]	Effect of probiotic supplementation on cognition and depressive symptoms in patients with depression: A systematic review and meta-analysis	10.1097/MD.0000000000036005	WOS + PubMed + Scopus	Different main outcome
Shefali et al. [39]	Effect of Probiotics on Mental Health and Their Association With Serum Neurometabolites in Adults With Depression or Anxiety: A Systematic Review and Meta-Analysis	10.14309/01.ajg.0000862800.37563.c2	WOS	Poster Presentation
Desai et al. [40]	Effectiveness of Probiotic, Prebiotic, and Synbiotic Supplementation to Improve Perinatal Mental Health in Mothers: A Systematic Review and Meta-Analysis	10.3389/fpsyt.2021.622181	WOS + PubMed	Perinatal Mental Health
Trifkovič et al. [41]	Efficacy of Direct or Indirect Use of Probiotics for the Improvement of Maternal Depression during Pregnancy and in the Postnatal Period: A Systematic Review and Meta-Analysis	10.3390/healthcare10060970	WOS + PubMed	Pregnancy and in the Postnatal
Dubreucq et al. [42]	Examining the evidence on complementary and alternative therapies to treat peripartum depression in pregnant or postpartum women: study protocol for an umbrella review of systematic reviews and meta-analyses	10.1136/bmjopen-2021-057327	Scopus	study protocol

Luo et al. [43]	Fermented dairy foods consumption and depressive symptoms: A meta-analysis of cohort studies	10.1371/journal.pone.0281346	WOS	cohort studies
Halemani et al. [44]	Impact of probiotic on anxiety and depression symptoms in pregnant and lactating women and microbiota of infants: A systematic review and meta-analysis	10.7189/jogh.13.04038	WOS + PubMed	Pregnant and lactating women
Sikorska et al. [45]	Probiotics as a Tool for Regulating Molecular Mechanisms in Depression: A Systematic Review and Meta-Analysis of Randomized Clinical Trials	10.3390/ijms24043081	WOS + PubMed + Scopus	Did not quantify symptoms
Dehghani et al. [46]	Probiotics supplementation and brain-derived neurotrophic factor (BDNF): a systematic review and meta-analysis of randomized controlled trials	10.1080/1028415X.2022.2110664	WOS + PubMed	Main Outcome Neurotrophin
Du et al. [47]	Probiotics/prebiotics/synbiotics and human neuropsychiatric outcomes: an umbrella review	10.1163/18762891-BJA00035	WOS + PubMed + Scopus	Umbrella Review
Scott et al. [48]	Systematic review and meta-analysis of augmentation and combination treatments for early-stage treatment-resistant depression	10.1177/02698811221104058	PubMed	Outcome of Pharmacological Therapies
Chen et al. [49]	Therapeutic effects of probiotics on symptoms of depression in children and adolescents: a systematic review and meta-analysis	10.1186/s13052-024-01807-6	PubMed + WOS + Scopus	symptoms of depression in children and adolescents
Un-Nisa et al. [50]	Updates on the Role of Probiotics against Different Health Issues: Focus on Lactobacillus.	10.3390/ijms24010142	EBSCO	Narrative Review
Ribeira et al. [51]	Probiotic, prebiotic, synbiotic and fermented food supplementation in psychiatric disorders: A systematic review of clinical trials	10.1016/j.neubiorev.2024.105561	Other sources	Systematic Review
Vaghef-Mehrabany et al. [52]	Can psychobiotics moodify gut? An update systematic review of randomized controlled trials in healthy and clinical subjects, on anti-depressant effects of probiotics, prebiotics, and synbiotics	10.1016/j.clnu.2019.06.004	WOS	Only Systematic
Sanada et al. [53]	Gut microbiota and major depressive disorder: A systematic review and meta-analysis	10.1016/j.jad.2020.01.102	WOS + PubMed + Scopus	Only Observacional
Chen et al. [54]	Pharmacological and non-pharmacological treatments for major depressive disorder in adults: A systematic review and network meta-analysis	10.1016/j.psychres.2019.112595	WOS + PubMed + Scopus	Network + Lack of data

Table S4. Methodological Quality Assessment of the Included Reviews Using the AMSTAR 2 Tool

Criteria	Huang et al. (2016)	Mckean et al. (2017)	Liu et al. (2018)	Reis et al. (2018)	Ng et al. (2018)	Liu et al. (2019)	Goh et al. (2019)	Nikolova et al. (2019)	Chen et al., 2019	Zagórska et al., (2020)	Chao et al., 2020	Zhang et al., 2020	Amirani et al., 2020	Hofmeister et al. (2021)	Nikolova et al. (2021)	Cohen Kadosh et al. (2021)	Misera et al., 2021	El Dib et al. (2021)	Zhu et al. (2022)	Le Morvan et al., 2022	Zhang et al. (2023)	Lin et al., (2023)	Zhao et al., 2023	Huang et al., 2024	Rahmannia et al., 2024	Asad et al. (2024)	Sulaiman et al., 2025	Zhao et al., 2025)	Zandifar et al., 2025	Cheng et al., 2025	Moshfeghinia et al., 2025
1. Questions and inclusion criteria – PICO	✓	✓	✓	✓	✓	✓	✓	✓	✓	✓	✓	✓	✓	✓	✓	✓	✓	✓	✓	✓	✓	✓	✓	✓	✓	✓	✓	✓	✓	✓	✓
2. Register or report justify any deviations from the protocol	✓	✗	✓	✓	✓	✗	✓	✗	✗	✗	✗	✓	✗	✓	✗	✗	✓	✓	✓	✓	✓	✓	✓	✗	✓	✓	✗	✓	✓	✗	✓
3. Selection of the study designs for inclusion in the review	✓	✓	✓	✓	✓	✓	✓	✓	✓	✓	✓	✓	✓	✓	✓	✓	✓	✓	✓	✓	✓	✓	✓	✓	✓	✓	✓	✓	✓	✓	✓
4. Comprehensive literature search strategy	✓	⚠	✓	✓	✓	✓	✓	⚠	⚠	✓	✓	✓	✓	✓	✓	✓	✓	✓	✓	✓	✓	✓	✓	✓	✓	✓	✓	✓	✓	✓	✓
5. Selection of duplicate studies	✓	⚠	✓	✓	✓	✓	✓	✓	✓	✓	✓	✗	✗	✓	✗	✓	✓	✓	✓	✓	✓	✓	✗	✓	✗	✓	✓	✓	✓	✓	✓
6. Duplicate data extraction	✓	⚠	✓	✓	✗	✓	✓	✓	✓	✗	✓	✗	✓	✓	✗	⚠	✗	✓	✓	✓	✓	✓	✓	✗	✓	✓	✓	✓	✓	✓	✓
7. List of excluded studies	✓	✗	✓	✓	✓	✓	✓	⚠	⚠	✓	⚠	⚠	✗	✓	✓	✓	✓	✓	✓	✓	✓	✓	✓	✓	⚠	✓	✓	✓	⚠	✓	✓
8. Description of the characteristics of the included studies	✓	✓	✓	✓	✓	✓	✓	✓	✓	✓	✓	✓	✓	✓	✓	✓	✓	✗	✓	✓	✓	✓	✓	✓	✓	✓	✓	✓	✗	✓	✓
9. Assessment of the risk of bias of individual studies	✓	⚠	✓	✓	✓	✓	✓	✓	✓	✓	✓	✓	⚠	✓	✓	✓	⚠	✓	✓	✓	✓	✓	✓	✓	✓	✓	✓	✓	⚠	✓	✓
10. Report of the source of funding for the included studies	⚠	⚠	⚠	⚠	⚠	⚠	⚠	⚠	⚠	⚠	⚠	⚠	⚠	✓	✓	⚠	✗	✓	✓	✓	✓	✓	⚠	⚠	⚠	✓	✓	⚠	⚠	✓	✓
11. Appropriate methods for meta-analysis	✓	✓	✓	✓	✓	✓	✓	✓	✓	✓	✓	✓	⚠	✓	✓	✓	⚠	✓	✓	✓	✓	✓	✓	✓	✓	✓	✓	✓	✓	✓	✓
12. Assessing the impact of risk of bias on results	✓	⚠	✓	✓	✓	✓	✓	✓	✓	✓	✓	✓	✗	✓	✓	✓	✓	✓	✓	✓	✓	✓	✓	✓	✓	✓	✓	✓	⚠	✓	✓
13. Assessment of statistical heterogeneity	✓	✓	✓	✓	✓	✓	✓	✓	✓	✓	✓	✓	✗	✓	✓	✓	✓	✓	✓	✓	✓	✓	✓	✓	✗	✓	✓	✗	⚠	✓	✓
14. Investigation of possible causes of heterogeneity	✓	✓	✓	✓	✓	✓	✓	✓	✗	✓	⚠	✓	✗	✓	✓	✓	✗	✓	✓	✓	✓	✓	✓	✗	✗	✓	✓	✗	⚠	✓	✓
15. Publication bias assessment	✓	⚠	✓	✓	✓	✓	✓	✓	✗	✓	✓	⚠	✗	✓	✓	✓	⚠	✓	✓	✓	✓	✓	✓	✓	✗	✓	✓	✓	⚠	✓	✓
16. Declaration of conflict of interest and funding	✓	✓	✓	✓	✓	✓	✓	✓	✓	✓	✗	✓	✓	✓	✓	✓	✓	✓	✓	✓	✓	✓	✓	✓	✓	✓	✓	✓	✗	✓	✓

✓ - Suitable Items; ✗ - Unattended Items; ⚠ - Partially Fulfilled Items;

Table S5. Justification for including SMD Global or SMD subgroup for analysis

Author [Number ref.]	SMD included	Justification for inclusion and analysis
**Depression**		
Huang et al., [1]	Global SMD	Subgroup: Only 1 evidence with a disorder is included, which would inflate the results.
Liu et al., [6]	Subgroup SMD	Specific trials for depression
Ng et al., [5]	Subgroup SMD	Specific individuals with mild-moderate depressive symptoms
Goh et al., [7]	Subgroup SMD	Specific individuals with mild-moderate depressive symptoms
Nikolova et al., [8]	Global SMD	Only the global SMD value is positioned.
Zagórska et al., [9]	Subgroup SMD	Specific individuals with mild-moderate depressive symptoms
Chao et al., [10]	Global SMD	The population studied consists specifically of individuals with depression and anxiety disorders.
Hofmeister et al., [13]	Subgroup SMD	Subgroup: Populations with depression (healthy excluded)
Nikolova et al., [14]	Global SMD	Only reports SMD Global and Subgroups without differentiation.
El Dib et al., [16]	Subgroup SMD	The author segments the SMD by subgroup of scales. The BDI scale showed the highest number of investigations.
Misera et al., [17]	Global SMD	Due to the small number of studies included
Zhu et al., [18]	Subgroup SMD	Subgroup: Populations with depression (healthy excluded)
Le Movan et al., [19]	Global SMD	SMD Global showcases 15 specific papers on depressive disorder.
Zhang et al., [20]	Subgroup SMD	SMD included based on the severity of depressive symptoms.
Lin et al., [21]	Global SMD	SMD is included based on the primary outcome (BDI scale) selected by the author. The DASS scale, defined as a secondary outcome
Huang et al., [23]	Subgroup SMD	Subgroup included with varying severity of depressive symptoms.
Asad et al., [25]	Subgroup SMD	SMD was included only for clinically diagnosed patients.
Zandifar et al., [28]	Global SMD	Only the global SMD value is positioned.
Cheng et al., [29]	Global SMD	Homogeneous population with depressive symptoms
Sulaiman et al., [26]	Global SMD	We are considering including the SMD containing all the scales analyzed. Segmentation drastically reduces the number of studies
Zhao et al., [27]	Global SMD	The population studied is homogeneous.
Moshfeghinia et al., [30]	Subgroup SMD	Subgroup included with varying severity of depressive symptoms.
**Anxiety**		
Liu et al., [3]	Global SMD	It does not report who the Unhealthy participants are.
Reis et al. [4]	Global SMD	Only available data
Liu et al., [6]	Subgroup SMD	Specific trials for anxiety - After outlier exclusion
Chao et al., [10]	Global SMD	The population studied consists specifically of individuals with depression and anxiety disorders.
Zhang et al., [11]	Subgroup SMD	stress-related subthreshold anxiety/depression level
Cohen Kadosh et al., [15]	Global SMD	We included SMD Global. The author does not explain the origin of the subgroup.
El Dib et al., [16]	Subgroup SMD	STAI scale subgroup was included because it is the most prevalent and consistent among different studies.
Le Movan et al., [19]	Global SMD	SMD Global showcases 17 specific papers on anxiety disorder.

Zhao et al., [22]	Subgroup SMD	Subgroups of individuals with mental health problems
Asad et al., [25]	Subgroup SMD	SMD was included only for clinically diagnosed patients.
Zandifar et al., [28]	Global SMD	Only the global SMD value is positioned.
Moshfeghinia et al., [30]	Global SMD	It segments the analysis by subgroup based on the severity of depression, but not specifically for anxiety.

REFERENCES

1. Huang R, Wang K, Hu J. Effect of Probiotics on Depression: A Systematic Review and Meta-Analysis of Randomized Controlled Trials. Nutrients. 2016;8(8). Epub 2016/08/12. doi: 10.3390/nu8080483. PubMed PMID: 27509521; PubMed Central PMCID: PMCPMC4997396.
2. McKean J, Naug H, Nikbakht E, Amiet B, Colson N. Probiotics and Subclinical Psychological Symptoms in Healthy Participants: A Systematic Review and Meta-Analysis. J Altern Complement Med. 2017;23(4):249-58. Epub 2016/11/15. doi: 10.1089/acm.2016.0023. PubMed PMID: 27841940.
3. Liu B, He Y, Wang M, Liu J, Ju Y, Zhang Y, et al. Efficacy of probiotics on anxiety-A meta-analysis of randomized controlled trials. Depress Anxiety. 2018;35(10):935-45. Epub 2018/07/12. doi: 10.1002/da.22811. PubMed PMID: 29995348.
4. Reis DJ, Ilardi SS, Punt SEW. The anxiolytic effect of probiotics: A systematic review and meta-analysis of the clinical and preclinical literature. PLoS One. 2018;13(6):e0199041. Epub 2018/06/21. doi: 10.1371/journal.pone.0199041. PubMed PMID: 29924822; PubMed Central PMCID: PMCPMC6010276.
5. Ng QX, Peters C, Ho CYX, Lim DY, Yeo WS. A meta-analysis of the use of probiotics to alleviate depressive symptoms. J Affect Disord. 2018;228:13-9. Epub 2017/12/05. doi: 10.1016/j.jad.2017.11.063. PubMed PMID: 29197739.
6. Liu RT, Walsh RFL, Sheehan AE. Prebiotics and probiotics for depression and anxiety: A systematic review and meta-analysis of controlled clinical trials. Neurosci Biobehav Rev. 2019;102:13-23. Epub 2019/04/21. doi: 10.1016/j.neubiorev.2019.03.023. PubMed PMID: 31004628; PubMed Central PMCID: PMCPMC6584030.
7. Goh KK, Liu YW, Kuo PH, Chung YE, Lu ML, Chen CH. Effect of probiotics on depressive symptoms: A meta-analysis of human studies. Psychiatry Res. 2019;282:112568. Epub 2019/09/30. doi: 10.1016/j.psychres.2019.112568. PubMed PMID: 31563280.
8. Nikolova V, Zaidi SY, Young AH, Cleare AJ, Stone JM. Gut feeling: randomized controlled trials of probiotics for the treatment of clinical depression: Systematic review and meta-analysis. Ther Adv Psychopharmacol. 2019;9:2045125319859963. Epub 2019/07/03. doi: 10.1177/2045125319859963. PubMed PMID: 31263542; PubMed Central PMCID: PMCPMC6595633.
9. Zagorska A, Marcinkowska M, Jamrozik M, Wisniowska B, Pasko P. From probiotics to psychobiotics - the gut-brain axis in psychiatric disorders. Benef Microbes. 2020;11(8):717-32. Epub 2020/11/17. doi: 10.3920/BM2020.0063. PubMed PMID: 33191776.
10. Chao L, Liu C, Sutthawongwadee S, Li Y, Lv W, Chen W, et al. Effects of Probiotics on Depressive or Anxiety Variables in Healthy Participants Under Stress Conditions or With a Depressive or Anxiety Diagnosis: A Meta-Analysis of Randomized Controlled Trials. Front Neurol. 2020;11:421. Epub 2020/06/13. doi: 10.3389/fneur.2020.00421. PubMed PMID: 32528399; PubMed Central PMCID: PMCPMC7257376.
11. Zhang N, Zhang Y, Li M, Wang W, Liu Z, Xi C, et al. Efficacy of probiotics on stress in healthy volunteers: A systematic review and meta-analysis based on randomized controlled trials. Brain Behav. 2020;10(9):e01699. Epub 2020/07/15. doi: 10.1002/brb3.1699. PubMed PMID: 32662591; PubMed Central PMCID: PMCPMC7507034.
12. Amirani E, Milajerdi A, Mirzaei H, Jamilian H, Mansournia MA, Hallajzadeh J, et al. The effects of probiotic supplementation on mental health, biomarkers of inflammation and oxidative stress in patients with psychiatric disorders: A systematic review and meta-analysis of randomized controlled trials. Complement Ther Med. 2020;49:102361. Epub 2020/03/10. doi: 10.1016/j.ctim.2020.102361. PubMed PMID: 32147043.
13. Hofmeister M, Clement F, Patten S, Li J, Dowsett LE, Farkas B, et al. The effect of interventions targeting gut microbiota on depressive symptoms: a systematic review and meta-analysis. CMAJ Open. 2021;9(4):E1195-E204. Epub 2021/12/23. doi: 10.9778/cmajo.20200283. PubMed PMID: 34933877; PubMed Central PMCID: PMCPMC8695538.
14. Nikolova VL, Cleare AJ, Young AH, Stone JM. Updated Review and Meta-Analysis of Probiotics for the Treatment of Clinical Depression: Adjunctive vs. Stand-Alone Treatment. J Clin Med. 2021;10(4). Epub 2021/02/12. doi: 10.3390/jcm10040647. PubMed PMID: 33567631; PubMed Central PMCID: PMCPMC7915600.

15. Cohen Kadosh K, Basso M, Knytl P, Johnstone N, Lau JYF, Gibson GR. Psychobiotic interventions for anxiety in young people: a systematic review and meta-analysis, with youth consultation. Transl Psychiatry. 2021;11(1):352. Epub 2021/06/17. doi: 10.1038/s41398-021-01422-7. PubMed PMID: 34131108; PubMed Central PMCID: PMCPMC8206413.
16. El Dib R, Periyasamy AG, de Barros JL, Franca CG, Senefonte FL, Vesentini G, et al. Probiotics for the treatment of depression and anxiety: A systematic review and meta-analysis of randomized controlled trials. Clin Nutr ESPEN. 2021;45:75-90. Epub 2021/10/09. doi: 10.1016/j.clnesp.2021.07.027. PubMed PMID: 34620373.
17. Misera A, Liskiewicz P, Loniewski I, Skonieczna-Zydecka K, Samochowiec J. Effect of Psychobiotics on Psychometric Tests and Inflammatory Markers in Major Depressive Disorder: Meta-Analysis of Randomized Controlled Trials with Meta-Regression. Pharmaceuticals (Basel). 2021;14(10). Epub 2021/10/24. doi: 10.3390/ph14100952. PubMed PMID: 34681176; PubMed Central PMCID: PMCPMC8541446.
18. Zhu H, Tian P, Zhao J, Zhang H, Wang G, Chen W. A psychobiotic approach to the treatment of depression: A systematic review and meta-analysis. Journal of Functional Foods. 2022;91(104999):1-7.
19. Le Morvan de Sequeira C, Hengstberger C, Enck P, Mack I. Effect of Probiotics on Psychiatric Symptoms and Central Nervous System Functions in Human Health and Disease: A Systematic Review and Meta-Analysis. Nutrients. 2022;14(3). Epub 2022/03/13. doi: 10.3390/nu14030621. PubMed PMID: 35276981; PubMed Central PMCID: PMCPMC8839125.
20. Zhang Q, Chen B, Zhang J, Dong J, Ma J, Zhang Y, et al. Effect of prebiotics, probiotics, synbiotics on depression: results from a meta-analysis. BMC Psychiatry. 2023;23(1):477. Epub 2023/06/30. doi: 10.1186/s12888-023-04963-x. PubMed PMID: 37386630; PubMed Central PMCID: PMCPMC10308754.
21. Lin J, Zhang Y, Wang K, Wang J, Kou S, Chen K, et al. The effect and safety of probiotics on depression: a systematic review and meta-analysis of randomized controlled trials. Eur J Nutr. 2023;62(7):2709-21. Epub 2023/05/29. doi: 10.1007/s00394-023-03184-y. PubMed PMID: 37247076.
22. Zhao Z, Xiao G, Xia J, Guo H, Yang X, Jiang Q, et al. Effectiveness of probiotic/prebiotic/synbiotic treatments on anxiety: A systematic review and meta-analysis of randomized controlled trials. J Affect Disord. 2023;343:9-21. Epub 2023/09/22. doi: 10.1016/j.jad.2023.09.018. PubMed PMID: 37734624.
23. Huang R, Liu Y. Efficacy of bifidobacterium-related preparations on depression: the first meta-analysis. Front Psychiatry. 2024;15:1463848. Epub 2024/10/18. doi: 10.3389/fpsyt.2024.1463848. PubMed PMID: 39421068; PubMed Central PMCID: PMCPMC11484414.
24. Rahmannia M, Poudineh M, Mirzaei R, Aalipour MA, Shahidi Bonjar AH, Goudarzi M, et al. Strain-specific effects of probiotics on depression and anxiety: a meta-analysis. Gut Pathog. 2024;16(1):46. Epub 2024/09/09. doi: 10.1186/s13099-024-00634-8. PubMed PMID: 39245752; PubMed Central PMCID: PMCPMC11382490.
25. Asad A, Kirk M, Zhu S, Dong X, Gao M. Effects of Prebiotics and Probiotics on Symptoms of Depression and Anxiety in Clinically Diagnosed Samples: Systematic Review and Meta-analysis of Randomized Controlled Trials. Nutr Rev. 2025;83(7):e1504-e20. Epub 2024/12/28. doi: 10.1093/nutrit/nuae177. PubMed PMID: 39731509; PubMed Central PMCID: PMCPMC12166186.
26. Sulaiman NNY, Mohamad Nizam NB, Mohd Noor NA, Lim SM, Ramasamy K, Alabsi AM, et al. An updated systematic review and appraisal of the pathophysiologic mechanisms of probiotics in alleviating depression. Nutr Neurosci. 2025:1-21. Epub 2025/07/16. doi: 10.1080/1028415X.2025.2531357. PubMed PMID: 40669008.
27. Zhao S, Liang S, Tao J, Peng Y, Chen S, Wai HKF, et al. Probiotics for adults with major depressive disorder compared with antidepressants: a systematic review and network meta-analysis. Nutr Rev. 2025;83(1):72-82. Epub 2024/01/14. doi: 10.1093/nutrit/nuad171. PubMed PMID: 38219239.
28. Zandifar A, Badrfam R, Mohammaditabar M, Kargar B, Goodarzi S, Hajialigol A, et al. The Effect of Prebiotics and Probiotics on Levels of Depression, Anxiety, and Cognitive Function: A Meta-Analysis of Randomized Clinical Trials. Brain Behav. 2025;15(3):e70401. Epub 2025/03/05. doi: 10.1002/brb3.70401. PubMed PMID: 40038860; PubMed Central PMCID: PMCPMC11879892.

29. Cheng Q, Ran Y, Mo X, Xiao R, He D, Guo S, et al. The efficacy and acceptability of Lactobacillus reuteri for the treatment of depression: A systematic review and meta-analysis. Gen Hosp Psychiatry. 2025;95:122-32. Epub 2025/05/09. doi: 10.1016/j.genhosppsych.2025.05.004. PubMed PMID: 40339531.
30. Moshfeghinia R, Nemati H, Ebrahimi A, Shekouh D, Karami S, Eraghi MM, et al. The impact of probiotics, prebiotics, and synbiotics on depression and anxiety symptoms of patients with depression: A systematic review and meta-analysis. J Psychiatr Res. 2025;188:104-16. Epub 2025/05/29 20:22. doi: 10.1016/j.jpsychires.2025.05.053. PubMed PMID: 40440772.
31. Budde H, Dolz N, Mueller-Alcazar A, Schacht F, Velasques B, Ribeiro P, et al. A 10 years update of effects of exercise on depression disorders-in otherwise healthy adults: A systematic review of meta-analyses and neurobiological mechanisms. PLoS One. 2025;20(5):e0317610. Epub 2025/05/05. doi: 10.1371/journal.pone.0317610. PubMed PMID: 40323978; PubMed Central PMCID: PMCPMC12052119.
32. Morán AC, Oba ER, Castañeda CD, Orozco AR, Loeza BM, Villalobos GG. Efficacy of Probiotics, Prebiotics, and Symbiotics for the Treatment of Depression: A meta-review. Salud Mental. 2025;48(1):31-46. doi: 10.17711/SM.0185-3325.2025.005.
33. Musazadeh V, Zarezadeh M, Faghfouri AH, Keramati M, Jamilian P, Jamilian P, et al. Probiotics as an effective therapeutic approach in alleviating depression symptoms: an umbrella meta-analysis. Crit Rev Food Sci Nutr. 2023;63(26):8292-300. Epub 2022/03/30. doi: 10.1080/10408398.2022.2051164. PubMed PMID: 35348020.
34. Goel R, Gupta B, Satodiya VN, Vala AU, Dabhi H, Mittal A. Association of Gut-Microbiome and mental health and effects of probiotics on psychiatric disorders: A Meta-analysis and systematic review. Niger Med J. 2025;66(1):13-25. doi: 10.71480/nmj.v66i1.601.
35. Breuling M, Tomeva E, Ivanovic N, Haslberger A. Butyrate- and Beta-Hydroxybutyrate-Mediated Effects of Interventions with Pro- and Prebiotics, Fasting, and Caloric Restrictions on Depression: A Systematic Review and Meta-Analysis. Life (Basel). 2024;14(7). Epub 2024/07/27. doi: 10.3390/life14070787. PubMed PMID: 39063542; PubMed Central PMCID: PMCPMC11278054.
36. Rokkas T, Ekmektzoglou K, Tsanou E, Bricca L, Menni AE, Golfakis P, et al. Comparative effectiveness and safety of probiotics with psychotropic potential in mental health benefits in irritable bowel syndrome: a systematic review and network meta-analysis. Eur J Gastroenterol Hepatol. 2025. Epub 2025/09/10. doi: 10.1097/MEG.0000000000003062. PubMed PMID: 40929652.
37. Haller H, Anheyer D, Cramer H, Dobos G. Complementary therapies for clinical depression: an overview of systematic reviews. BMJ Open. 2019;9(8):e028527. Epub 2019/08/07. doi: 10.1136/bmjopen-2018-028527. PubMed PMID: 31383703; PubMed Central PMCID: PMCPMC6686993.
38. He J, Chang L, Zhang L, Wu W, Zhuo D. Effect of probiotic supplementation on cognition and depressive symptoms in patients with depression: A systematic review and meta-analysis. Medicine (Baltimore). 2023;102(47):e36005. Epub 2023/11/28. doi: 10.1097/MD.0000000000036005. PubMed PMID: 38013351; PubMed Central PMCID: PMCPMC10681621.
39. Shefali G, Mai H, Laila A, Emerson F, Yen R, Emeny R. S1540 Effect of Probiotics on Mental Health and Their Association With Serum Neurometabolites in Adults With Depression or Anxiety: A Systematic Review and Meta-Analysis. The American Journal of Gastroenterology. 2022;117((10S)):e1102. doi: 10.14309/01.ajg.0000862800.37563.c2.
40. Desai V, Kozyrskyj AL, Lau S, Sanni O, Dennett L, Walter J, et al. Effectiveness of Probiotic, Prebiotic, and Synbiotic Supplementation to Improve Perinatal Mental Health in Mothers: A Systematic Review and Meta-Analysis. Front Psychiatry. 2021;12:622181. Epub 2021/05/11. doi: 10.3389/fpsyt.2021.622181. PubMed PMID: 33967849; PubMed Central PMCID: PMCPMC8100186.
41. Trifkovic KC, Micetic-Turk D, Kmetec S, Strauss M, Dahlen HG, Foster JP, et al. Efficacy of Direct or Indirect Use of Probiotics for the Improvement of Maternal Depression during Pregnancy and in the Postnatal Period: A Systematic Review and Meta-Analysis. Healthcare (Basel). 2022;10(6). Epub 2022/06/25. doi: 10.3390/healthcare10060970. PubMed PMID: 35742022; PubMed Central PMCID: PMCPMC9223194.

42. Dubreucq J, Kamperman AM, Al-Maach N, Bramer WM, Pacheco F, Ganho-Avila A, et al. Examining the evidence on complementary and alternative therapies to treat peripartum depression in pregnant or postpartum women: study protocol for an umbrella review of systematic reviews and meta-analyses. BMJ Open. 2022;12(11):e057327. Epub 2022/11/22. doi: 10.1136/bmjopen-2021-057327. PubMed PMID: 36410814; PubMed Central PMCID: PMCPMC9680168.
43. Luo Y, Li Z, Gu L, Zhang K. Fermented dairy foods consumption and depressive symptoms: A meta-analysis of cohort studies. PLoS One. 2023;18(2):e0281346. Epub 2023/02/07. doi: 10.1371/journal.pone.0281346. PubMed PMID: 36745637; PubMed Central PMCID: PMCPMC9901789.
44. Halemani K, Shetty AP, Thimmappa L, Issac A, Dhiraaj S, Radha K, et al. Impact of probiotic on anxiety and depression symptoms in pregnant and lactating women and microbiota of infants: A systematic review and meta-analysis. J Glob Health. 2023;13:04038. Epub 2023/05/23. doi: 10.7189/jogh.13.04038. PubMed PMID: 37218177; PubMed Central PMCID: PMCPMC10173681 Form (available upon request from the corresponding author) and disclose no relevant interests.
45. Sikorska M, Antosik-Wojcinska AZ, Dominiak M. Probiotics as a Tool for Regulating Molecular Mechanisms in Depression: A Systematic Review and Meta-Analysis of Randomized Clinical Trials. Int J Mol Sci. 2023;24(4). Epub 2023/02/26. doi: 10.3390/ijms24043081. PubMed PMID: 36834489; PubMed Central PMCID: PMCPMC9963932.
46. Dehghani F, Abdollahi S, Shidfar F, Clark CCT, Soltani S. Probiotics supplementation and brain-derived neurotrophic factor (BDNF): a systematic review and meta-analysis of randomized controlled trials. Nutr Neurosci. 2023;26(10):942-52. Epub 2022/08/24. doi: 10.1080/1028415X.2022.2110664. PubMed PMID: 35996352.
47. Du Q, Li Q, Liu C, Liao G, Li J, Yang J, et al. Probiotics/prebiotics/synbiotics and human neuropsychiatric outcomes: an umbrella review. Benef Microbes. 2024;15(6):589-608. Epub 2024/09/07. doi: 10.1163/18762891-bja00035. PubMed PMID: 39242081.
48. Scott F, Hampsey E, Gnanapragasam S, Carter B, Marwood L, Taylor RW, et al. Systematic review and meta-analysis of augmentation and combination treatments for early-stage treatment-resistant depression. J Psychopharmacol. 2023;37(3):268-78. Epub 2022/07/22. doi: 10.1177/02698811221104058. PubMed PMID: 35861202; PubMed Central PMCID: PMCPMC10076341.
49. Chen CM, Liang SC, Sun CK, Cheng YS, Tang YH, Liu C, et al. Therapeutic effects of probiotics on symptoms of depression in children and adolescents: a systematic review and meta-analysis. Ital J Pediatr. 2024;50(1):239. Epub 2024/11/13. doi: 10.1186/s13052-024-01807-6. PubMed PMID: 39511589; PubMed Central PMCID: PMCPMC11545225.
50. Un-Nisa A, Khan A, Zakria M, Siraj S, Ullah S, Tipu MK, et al. Updates on the Role of Probiotics against Different Health Issues: Focus on Lactobacillus. Int J Mol Sci. 2022;24(1). Epub 2023/01/09. doi: 10.3390/ijms24010142. PubMed PMID: 36613586; PubMed Central PMCID: PMCPMC9820606.
51. Ribera C, Sanchez-Orti JV, Clarke G, Marx W, Morkl S, Balanza-Martinez V. Probiotic, prebiotic, synbiotic and fermented food supplementation in psychiatric disorders: A systematic review of clinical trials. Neurosci Biobehav Rev. 2024;158:105561. Epub 2024/01/28. doi: 10.1016/j.neubiorev.2024.105561. PubMed PMID: 38280441.
52. Vaghef-Mehrabany E, Maleki V, Behrooz M, Ranjbar F, Ebrahimi-Mameghani M. Can psychobiotics "mood" ify gut? An update systematic review of randomized controlled trials in healthy and clinical subjects, on anti-depressant effects of probiotics, prebiotics, and synbiotics. Clin Nutr. 2020;39(5):1395-410. Epub 2019/07/17. doi: 10.1016/j.clnu.2019.06.004. PubMed PMID: 31307840.
53. Sanada K, Nakajima S, Kurokawa S, Barcelo-Soler A, Ikuse D, Hirata A, et al. Gut microbiota and major depressive disorder: A systematic review and meta-analysis. J Affect Disord. 2020;266:1-13. Epub 2020/02/15. doi: 10.1016/j.jad.2020.01.102. PubMed PMID: 32056863.
54. Chen C, Shan W. Pharmacological and non-pharmacological treatments for major depressive disorder in adults: A systematic review and network meta-analysis. Psychiatry Res. 2019;281:112595. Epub 2019/10/19. doi: 10.1016/j.psychres.2019.112595. PubMed PMID: 31627074.
