# Supplementary material for: “Attacking” the Gut–Brain Axis with Psychobiotics: An Umbrella Review of Depressive and Anxiety Symptoms
Source: Pharmaceuticals (Basel). 2026 Jan 15;19(1):156. doi: 10.3390/ph19010156 (PMC12845323; doi:10.3390/ph19010156)
Supplement: Supplementary file 1 [file pharmaceuticals-19-00156-s001.zip › pharmaceuticals-4039435-supplementary 2-PRISMA Checklist.pdf]

PRISMA

PRISMA 2020 Checklist

Section and Topic	Item #	Checklist item	Report
**TITLE**			
Title	1	Identify the report as a systematic review.	"An Umbrella Review and Critical Appraisal of Systematic reviews and Meta-Analyses" (p. 1)
**ABSTRACT**			
Abstract	2	See the PRISMA 2020 for Abstracts checklist.	Properly reported. The abstract of the manuscript provides aims, methods, and main findings. (p. 1)
**INTRODUCTION**			
Rationale	3	Describe the rationale for the review in the context of existing knowledge.	"Despite the multiplicity of published reviews, there remains a lack of integrative and comparative analyses across studies… Consequently, the field remains open to a study that not only consolidates the current state of the art but also rigorously and critically evaluates the methodological quality and overall landscape of existing reviews." Location: p. 2, lines 87–92
Objectives	4	Provide an explicit statement of the objective(s) or question(s) the review addresses.	"Therefore, the present study aims to systematically compile, analyze, and compare the main systematic reviews with meta-analyses on the use of psychobiotics in the treatment of depressive and anxiety disorder or symptom levels…" Location: p. 2, lines 92–97
**METHODS**			
Eligibility criteria	5	Specify the inclusion and exclusion criteria for the review and how studies were grouped for the syntheses.	"Only systematic reviews with meta-analyses of randomized controlled trials (RCTs) were eligible for inclusion. To be included, the trials had to investigate the effects of psychobiotic interventions, such as probiotics, prebiotics, or synbiotics, administered with the aim of improving symptoms of depression and/or anxiety and/or stress. Secondarily, changes in biological markers, such as neuroinflammatory indicators, were also examined. Only studies conducted in humans and published in English were considered." Location: p. 24, lines 741–754
Information sources	6	Specify all databases, registers, websites, organisations, reference lists and other sources searched or consulted to identify studies. Specify the date when each source was last searched or consulted.	"Between May and June 2025, two reviewers (JOF and ASF) independently conducted a comprehensive literature search using the PubMed/MEDLINE, Web of Science, Scopus, Scielo, EBSCO, and Cochrane Database electronic databases. The search was restricted to studies involving human subjects and published in English up to September 2025". Location: p. 25, lines 771–774
Search strategy	7	Present the full search strategies for all databases, registers and websites, including any filters and limits used.	The complete search strategy combined terms using Boolean operators across the different databases, as presented in Table S1 (supplementary material). Additionally, the file related to the extraction of all databases was deposited in The Open Science Framework - OSF (https://osf.io/10.17605/OSF.IO/FQ5C9). In general, we combined the following keywords, which varied in their inclusion format depending on the database: (psychobiotics OR probiotics OR prebiotics OR synbiotics) AND (depressive OR depressive disorder OR anxiety) AND meta-analysis. Location: p. 25, lines 780–782
Selection process	8	Specify the methods used to decide whether a study met the inclusion criteria of the review, including how many reviewers screened each record and each report retrieved, whether they worked independently, and if applicable, details of automation tools used in the process.	"The screening of titles, abstracts, and full texts was independently performed by two reviewers, strictly following the predefined inclusion and exclusion criteria. To assess the reliability of the selection process, Cohen's Kappa coefficient was calculated, which measures the degree of agreement beyond that expected by chance."<br>Location: p. 26, lines 841–844
Data collection process	9	Specify the methods used to collect data from reports, including how many reviewers collected data from each report, whether they worked independently, any processes for obtaining or confirming data from study investigators, and if applicable, details of automation tools used in the process.	"Two reviewers independently screened studies, extracted data, and evaluated methodological quality. Additional bibliometric, conceptual, and psychometric features were mapped, including geographical origin, publication timeline, scale distribution, and citation-based connectivity."<br>Location: p. 26, lines 830–832
Data items	10a	List and define all outcomes for which data were sought. Specify whether all results that were compatible with each outcome	"The primary outcomes of this review included changes in symptoms of depression and/or anxiety, assessed through validated psychometric instruments. All scales available in the

Section and Topic	Item #	Checklist item	Report
		domain in each study were sought (e.g. for all measures, time points, analyses), and if not, the methods used to decide which results to collect.	different meta-analyses were included, such as, Beck Depression Inventory (BDI), Depression, Anxiety and Stress Scales (DASS), Hospital Anxiety and Depression Scale (HADS)…" Location: p. 25, lines 785–794
	10b	List and define all other variables for which data were sought (e.g. participant and intervention characteristics, funding sources). Describe any assumptions made about any missing or unclear information.	"In addition to the traditional methodological variables, an expanded set of bibliometric and psychometric characteristics was systematically extracted to map temporal, geographical, and conceptual patterns across the included studies. Information regarding the geographical origin of each study (country and continent of conduct) was recorded to identify the spatial distribution of scientific production and potential regional asymmetries. Subsequently, all studies were organized according to their chronological order of publication, from which a timeline was constructed to illustrate the historical evolution of the evidence and periods of increased or reduced research activity. To characterize the conceptual structure of the literature, a citation-based connectivity map was generated capturing the structural relationships among the included studies, revealing variations in citation density and the strength of inter-study links…". Location: p. 25, lines 809–821
Study risk of bias assessment	11	Specify the methods used to assess risk of bias in the included studies, including details of the tool(s) used, how many reviewers assessed each study and whether they worked independently, and if applicable, details of automation tools used in the process.	"The methodological quality of the included systematic reviews was assessed using the AMSTAR 2 instrument. Two assessors independently carried out this process (JOF and ASF). AMSTAR 2 comprises 16 items addressing key methodological aspects such as the presence of a pre-registered protocol, adequacy of inclusion criteria, search strategy, assessment of risk of bias in primary studies, and appropriateness of statistical analyses. This tool does not generate a final numerical score but rather classifies the overall confidence in the review's findings as high, moderate, low, or critically low, allowing for a more qualitative interpretation of the methodological robustness of each evaluated study [72]." Location: p. 26, lines 830–837
Effect measures	12	Specify for each outcome the effect measure(s) (e.g. risk ratio, mean difference) used in the synthesis or presentation of results.	"Statistical analyses were conducted by extracting effect sizes as SMDs accompanied by their 95% confidence intervals (95%CI). Between-study variability was quantified using the I^2 statistic. The overall pooled effect was estimated using a random-effects model." Location: p. 26, lines 853–869
Synthesis methods	13a	Describe the processes used to decide which studies were eligible for each synthesis (e.g. tabulating the study intervention characteristics and comparing against the planned groups for each synthesis (item #5)).	"To minimize analytical heterogeneity and avoid distorting the behavior of the pooled estimates, the results were synthesized separately for depression scores and anxiety scores. This decision was based on the markedly larger number of studies evaluating the effects of probiotics on depressive symptoms compared to those assessing anxiety, which could have disproportionately influenced the overall summary effect if analyzed jointly." Location: p. 14, lines 234–246
	13b	Describe any methods required to prepare the data for presentation or synthesis, such as handling of missing summary statistics, or data conversions.	"In addition, corrections were applied to the directionality of the reported outcomes. Specifically, several meta-analyses presented positive SMDs to indicate improvements associated with probiotic supplementation. Because the present synthesis required all outcomes to be plotted on a common axis for comparability, these SMDs were systematically reoriented to negative values. This restandardization ensured conceptual consistency, whereby negative values uniformly reflected symptom improvement, and prevented misleading interpretations that could arise from mixing opposing effect directions within the same analytical framework. When individual studies reported mean differences rather than SMD, the values were systematically converted to SMD to ensure comparability across meta-analyses and maintain scale-independent interpretation." Location: p. 14, lines 234–245
	13c	Describe any methods used to tabulate or visually display results of individual studies and syntheses.	"Subsequently, all studies were organized according to their chronological order of publication, from which a timeline was constructed to illustrate the historical evolution of the evidence and periods of increased or reduced research activity. To characterize the conceptual structure of

Section and Topic	Item #	Checklist item	Report
			the literature, a citation-based connectivity map was generated capturing the structural relationships among the included studies, revealing variations in citation density and the strength of inter-study links. … Additionally, standardized extraction of the psychometric scales used in each study was performed … All scales were categorized according to their purpose, psychometric properties, and frequency of use, enabling comparative synthesis across instruments and identification of methodological convergences among the included studies." "General characteristics of the 33 eligible meta-analyses are summarized in Table 1." Location: p. 25, lines 814–819
	13d	Describe any methods used to synthesize results and provide a rationale for the choice(s). If meta-analysis was performed, describe the model(s), method(s) to identify the presence and extent of statistical heterogeneity, and software package(s) used.	"Stability of the pooled SMD across iterations was taken as evidence that the findings were not driven by isolated data points or outlier effect sizes. Cochran's Q test was used to assess the presence of heterogeneity, while the magnitude of inconsistency was quantified using the I^2 statistic, interpreted according to established thresholds (25% low, 50% moderate, and 75% high heterogeneity). In addition, the between-study variance (τ^2) was estimated using the DerSimonian–Laird method to account for the dispersion of true effect sizes across studies. Because substantial heterogeneity was expected due to differences in population characteristics, psychometric scales, intervention duration, probiotic strains, and study design, all pooled estimates were computed using random-effects models." Location: p. 26, lines 860–868
	13e	Describe any methods used to explore possible causes of heterogeneity among study results (e.g. subgroup analysis, meta-regression).	"Cochran's Q test was used to assess the presence of heterogeneity, while the magnitude of inconsistency was quantified using the I^2 statistic, interpreted according to established thresholds (25% low, 50% moderate, and 75% high heterogeneity)". Location: p. 26, lines 862–864
	13f	Describe any sensitivity analyses conducted to assess robustness of the synthesized results.	"Additionally, sensitivity analyses were carried out to assess the robustness of the findings by sequentially excluding studies judged to have a high variability". Location: p. 26, lines 862–864
Reporting bias assessment	14	Describe any methods used to assess risk of bias due to missing results in a synthesis (arising from reporting biases).	Not required for Umbrella review
Certainty assessment	15	Describe any methods used to assess certainty (or confidence) in the body of evidence for an outcome.	Not required for Umbrella review
**RESULTS**			
Study selection	16a	Describe the results of the search and selection process, from the number of records identified in the search to the number of studies included in the review, ideally using a flow diagram.	"The systematic search initially identified 529 articles related to the topic. After removing duplicates and reading by title and abstract, the screening observed 55 systematic reviews with meta-analyses across the selected databases and additional sources. Of these, 22 were excluded for not meeting the predefined inclusion criteria […], leaving 33 articles for full-text assessment. In the end, only 30 systematic reviews with meta-analyses remained. Figure 1 details the inclusion and exclusion process for selected articles. The supplementary material (Table S1) details the reasons for exclusion." Location: p. 2, lines 100–105
	16b	Cite studies that might appear to meet the inclusion criteria, but which were excluded, and explain why they were excluded.	"The supplementary material (Table S2). List of excluded articles and their reasons."
Study characteristics	17	Cite each included study and present its characteristics.	"General characteristics of the 33 eligible meta-analyses are summarized in Table 1. Probiotics accounted for the largest proportion, with 30 studies (100%) examining their effects on psychological outcomes. Prebiotics represented seven studies (23.3%), reflecting a smaller body of evidence focusing on isolated modulation of fermentable fibers. Synbiotics were evaluated in 4 studies (13.3%), indicating more limited interventions". Location: p. 6, lines 200–205

PRISMA

PRISMA 2020 Checklist

Section and Topic	Item #	Checklist item	Report
Risk of bias in studies	18	Present assessments of risk of bias for each included study.	“The methodological appraisal using the AMSTAR 2 framework revealed substantial variability in the confidence attributed to the included systematic reviews and meta-analyses, highlighting marked heterogeneity in their overall robustness. Among the 31 reviews assessed, only twelve (38.7%) achieved a rating of high confidence characterized by the absence of critical flaws and by strong adherence to core methodological standards, particularly comprehensive search strategies, rigorous risk-of-bias assessments, transparent synthesis protocols and registration in the PROSPERO database or similar. We highlight that, despite the generally high adherence to methodological quality criteria and the absence of critical flaws, some reviews exhibited shortcomings in adequately reporting specific AMSTAR 2 checklist items. Consequently, their overall compliance did not reach 100%”. Location: p. 19, lines 463–472
Results of individual studies	19	For all outcomes, present, for each study: (a) summary statistics for each group (where appropriate) and (b) an effect estimates and its precision (e.g. confidence/credible interval), ideally using structured tables or plots.	“Across the 30 included meta-analytic estimates, probiotics demonstrated the most consistent evidence of benefit for depressive and anxiety symptoms. For depressive outcomes, 92.0% of the probiotic meta-analytic estimates reported statistically significant reductions in symptom severity, although the effects appear somewhat inconsistent when analyzing other scales, such as MADRS. Positive effects were observed in the majority of studies”. Location: p. 14, 282–286
Results of syntheses	20a	For each synthesis, briefly summarise the characteristics and risk of bias among contributing studies.	“The included meta-analyses showed considerable methodological heterogeneity, both regarding search strategies and eligibility criteria, population, strains used in the intervention, as well as in relation to the outcomes analyzed.”
	20b	Present results of all statistical syntheses conducted. If meta-analysis was done, present for each the summary estimate and its precision (e.g. confidence/credible interval) and measures of statistical heterogeneity. If comparing groups, describe the direction of the effect.	“A total of 25 meta-analytic estimates reporting depressive symptom outcomes were synthesized, following a structured subgroup strategy restricted to patients with MDD or clinical conditions in which depressive symptoms were a primary or secondary diagnostic component. This approach ensured that the pooled effect specifically reflected populations in whom depressive symptomatology was clinically relevant, rather than aggregated data from heterogeneous or asymptomatic samples. The overall pooled effect demonstrated a moderate and statistically significant reduction in depressive symptoms in favor of probiotics (SMD = –0.47; 95% CI = –0.55 to –0.39, p = 0.001). This magnitude corresponds to a clinically meaningful improvement and was consistently observed across the subgroup of individuals with clinically defined depressive symptomatology”. Location: p. 15, lines 328–337
	20c	Present results of all investigations of possible causes of heterogeneity among study results.	“Only five meta-analyses (16.1%) demonstrated low heterogeneity ($I^2 \leq 25\%$) […] Moderate heterogeneity ($I^2 = 25\%$ to 50%) was observed in five studies (16.1%) […] the largest proportion of meta-analyses fell within the category of substantial heterogeneity ($I^2 = 50\%$ to 75%), comprising nine studies (29.0%).”
	20d	Present results of all sensitivity analyses conducted to assess the robustness of the synthesized results.	“The sensitivity analysis demonstrated a high degree of stability in the estimates. Excluding any individual study resulted in only marginal changes in the pooled SMD, which ranged from –0.48 to –0.44. Notably, none of the recalculated models yielded confidence intervals crossing zero, indicating that the effect remained statistically significant under all evaluated conditions. Studies with more extreme SMDs (e.g., Moshfeghinia et al., [51], SMD = –1.22) or smaller effect sizes (e.g., Liu et al., [28], SMD = –0.12) did not exert disproportionate influence on the overall result. The minimal variation observed (<0.04 points in the pooled SMD) indicates that both the direction and magnitude of the effect are consistent and not driven by any single study”. Location: p. 16, lines 347–354
Reporting biases	21	Present assessments of risk of bias due to missing results (arising from reporting biases) for each synthesis assessed.	“The most frequent shortcomings included […] omission of publication bias assessment (Item 15). These recurrent methodological gaps suggest that a substantial portion of available syntheses provide limited reliability for decision-making […].”
Certainty of evidence	22	Present assessments of certainty (or confidence) in the body of evidence for each outcome assessed.	Not required for Umbrella review

PRISMA

PRISMA 2020 Checklist

Section and Topic	Item #	Checklist item	Report
**DISCUSSION**			
Discussion	23a	Provide a general interpretation of the results in the context of other evidence.	"This umbrella review synthesized and critically appraised 30 systematic reviews and meta-analyses evaluating the effects of psychobiotic interventions on depressive and anxiety symptoms in adults with a confirmed diagnosis or with symptoms consistent with the disorders. Overall, the evidence indicates that probiotics demonstrate moderate and consistent beneficial effects on depressive symptoms, whereas findings for anxiety, they show only minor effects. Additionally, in the context of prebiotics, only a significant, but marginal, effect was observed for symptoms of depression, whereas for anxiety, the body of evidence available in the literature is poor. Supplementation with synbiotics remain limited. These results align with previous evidence suggesting that gut–brain modulation may influence affective regulation". Location: p. 21, lines 538–546
	23b	Discuss any limitations of the evidence included in the review.	"During the process of collecting and synthesizing the various meta-analyses, we identified potential sources of confusion, including the directionality of reported outcomes in some studies. When evaluating effects based on psychometric instruments such as the BDI, HAM-D, or DASS, one expects results to be expressed as reductions in symptom scores, given that lower scores correspond to clinical improvement. Consequently, when reporting effect estimates, the direction of the effect should mathematically be negative (post-intervention values must be lower than baseline for the intervention to be interpreted as beneficial)". Location: p. 23, lines 666–572
	23c	Discuss any limitations of the review processes used.	Limitations added at different points in the discussion. "In other hands, our findings in the context of probiotic supplementation to mitigate the effects of anxiety, do not appear to be substantially reliable (SMD = –0.34; 95% CI: –0.47 to –0.21; $p < 0.001$), since elevated levels of anxiety, i.e., an exaggerated, transient, and functional response, do not constitute an anxiety disorder, a sustained neurobiological dysfunction accompanied by functional impairment". Location: p. 22, lines 612–614
	23d	Discuss implications of the results for practice, policy, and future research.	"For now, we may regard these findings as a promising indicator for future experiments targeting anxiety disorders".
**OTHER INFORMATION**			
Registration and protocol	24a	Provide registration information for the review, including register name and registration number, or state that the review was not registered.	"number CRD420251164884".<br>Location: p. 22, lines 737–738
	24b	Indicate where the review protocol can be accessed, or state that a protocol was not prepared.	"PROSPERO database<br>Location: p. 22, lines 737–738
	24c	Describe and explain any amendments to information provided at registration or in the protocol.	No changes were made.
Support	25	Describe sources of financial or non-financial support for the review, and the role of the funders or sponsors in the review.	"This research received no external funding."
Competing interests	26	Declare any competing interests of review authors.	"Conflicts of Interest: The authors declare no conflict of interest."
Availability of data, code and other materials	27	Report which of the following are publicly available and where they can be found: template data collection forms; data extracted from included studies; data used for all analyses; analytic code; any other materials used in the review.	"All data used in this study are publicly available from published articles cited within this manuscript. The complete dataset and analysis code are available upon reasonable request from the corresponding author."
